# Two New Metabolites from *Moquiniastrum sordidum*: Phytochemical Characterization and Multifaceted Bioactivity

**DOI:** 10.1021/acsomega.6c06069

**Published:** 2026-10-20

**Authors:** Tamires Cordeiro, Francielli A. P. Valeze, Rodolfo B. Balbinot, Bianca D. B. Sahm, Fernanda F. Dourado, Elizabeth F. Pires, Aline Pivetta, Tatiana S. Tiuman, Celso V. Nakamura, Leticia V. Costa-Lotufo, Marta R. B. do Carmo, Renan V. Viesser, Ernani A. Basso, Maria H. Sarragiotto, Debora C. Baldoqui

**Affiliations:** † Departamento de Química, 42487 Universidade Estadual de Maringá, Av. Colombo 5790, Maringá 87020-900, PR, Brazil; ‡ Programa de Pós-Graduação em Ciências Biológicas, Universidade Estadual de Maringá, Av. Colombo 5790, 87020-900 Maringá, Paraná, Brazil; § Instituto de Ciências Biomédicas, Universidade de São Paulo, Av. Prof. Lineu Prestes, 1524 São Paulo, SP, Brazil; ∥ Universidade Tecnológica Federal do Paraná, R. Cristo Rei, 19, 85902-490 Toledo, PR, Brazil; ⊥ Departamento de Biologia Geral, Universidade Estadual de Ponta Grossa, Av. Carlos Cavalcanti, 4748 Ponta Grossa, PR, Brazil

## Abstract

Recent taxonomic revisions based on molecular phylogenetics transferred species from *Gochnatia* to *Moquiniastrum*, which now includes 22 species, 20 of which occur in Brazil with an extra-Amazonian distribution. Here, we report a phytochemical study of *Moquiniastrum sordidum* (Less.) G. Sancho. Chromatographic separation afforded eight compounds, including two new metabolites: a sesquiterpene (**5**) and a phenolic glycoside (**8**). The known compounds were identified as simiarenol (**1**), oleanolic acid (**2**), hispidulin (**3**), tyrosol (**4**), genkwanin (**6**), and apigenin (**7**). The relative configuration of compound **5** was established using *pip*-HSQMBC-IPAP NMR, which afforded accurate long-range ^2,3^ *J*_CH_ coupling constants. Dereplication enabled the putative annotation of 45 metabolites: 12 flavonoids, 10 chlorogenic acid derivatives, 5 hydroxycinnamic acid derivatives, 9 terpenes, and 9 others. The biological potential of the extracts and isolated metabolites was evaluated using antioxidant, cytotoxic, antimicrobial, and antileishmanial assays. The new phenolic glycoside (**8**) showed potent antioxidant activity (ABTS: 1111 μmol TE g^–1^; FRAP: 4293 μmol Fe­(II) g^–1^). Oleanolic acid (MIC = 130 μg mL^–1^) and apigenin (MIC = 30 μg mL^–1^) showed activity against the Gram-positive bacterium *Bacillus cereus*. Preliminary cytotoxic screening against HCT-116 cells showed that the dichloromethane fraction inhibited proliferation by 92% at 50 μg mL^–1^. Antileishmanial evaluation revealed moderate activity of the crude extract, which was significantly enhanced upon fractionation, with the dichloromethane fraction showing the highest activity against intracellular amastigotes (IC_50_ = 8.23 μg mL^–1^; SI = 7.87), indicating a selective antiparasitic effect.

## Graphical abstract

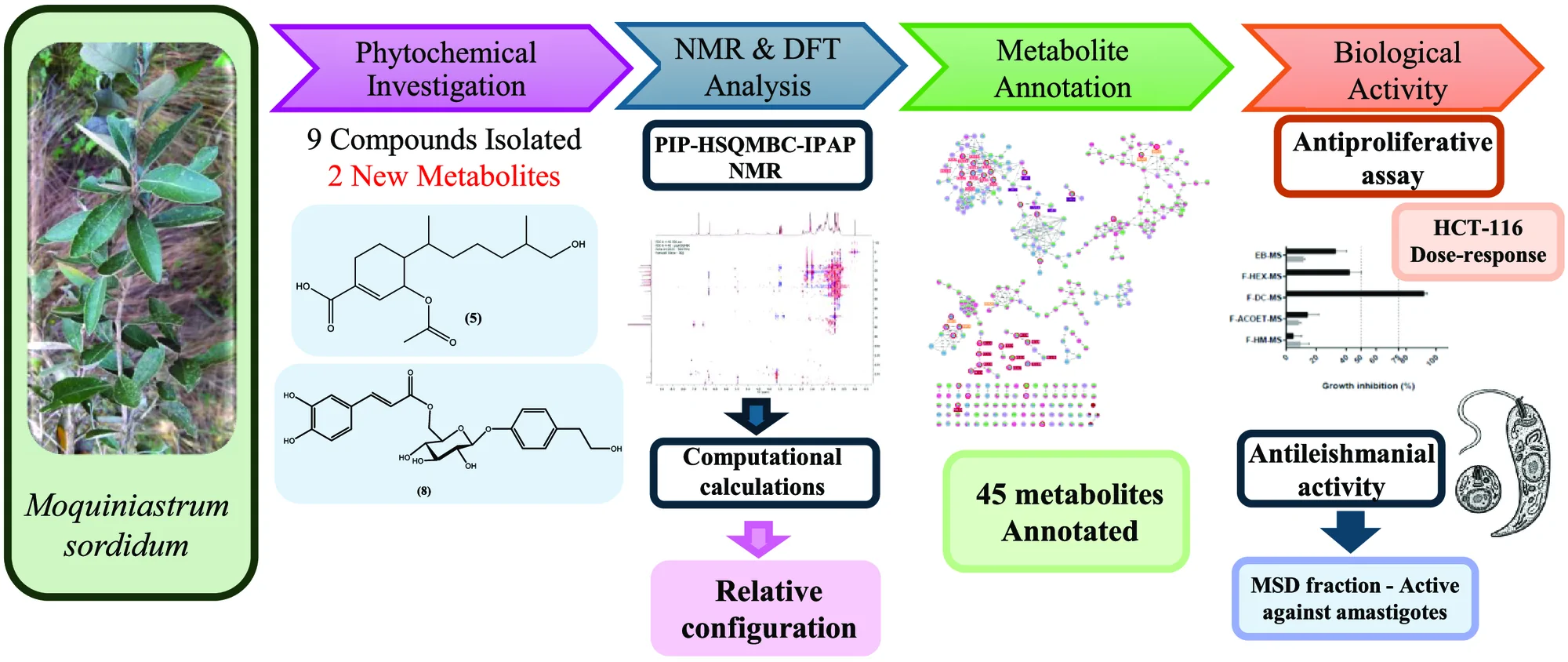

## 1 Introduction

Taxonomic revisions within the tribe Gochnatieae have led to the reclassification of several species previously recognized within the paraphyletic genus *Gochnatia* into the new genus *Moquiniastrum*.^1^ This decision is primarily supported by molecular phylogenetic analyses, which revealed that these species form a distinct and well-supported clade, justifying the recognition of *Moquiniastrum* as an independent genus.^1^ The application of advanced phylogenomic tools has provided greater clarity at the genus level, confirming that the transferred species are more accurately placed within *Moquiniastrum* rather than *Gochnatia*.^2^

The genus *Moquiniastrum* comprises 22 recognized species distributed across South America, including Argentina, Bolivia, Paraguay, Peru, Uruguay, and Venezuela, with significant diversity in Brazil.^3^ Twenty species are documented in Brazil, 13 of which are endemic, with an extra-Amazonian distribution.^3,4^

The most studied species in this genus is *Moquiniastrum polymorphum* (formerly *Gochnatia polymorpha*), which is a rich source of bioactive specialized metabolites, including sesquiterpene lactones, *ent*-kaurene diterpenes, triterpenes, flavonoids, and coumarins.^5–8^ Similar classes of metabolites, including chlorogenic acid derivatives and flavones with strong antiradical activity, have been reported in *Myoporum floribundum* (formerly *Gochnatia floribunda*).^9–11^ Additionally, *Gochnatia pulchra* contains dimeric guaianolides and other compounds with antimicrobial and cytotoxic properties, reinforcing the genus as a promising source of therapeutic agents.^12,13^

Continuing our investigation of Asteraceae species occurring in the Campos Gerais region of Paraná State (southern Brazil), herein we present a phytochemical study of *M. sordidum* (Less.) G. Sancho, a shrub species threatened with extinction and classified as “vulnerable.”^14^ From the aerial parts of this species, two novel compounds were isolated alongside six known compounds, predominantly methoxylated flavonoids. In addition, dereplication analysis using ultrahigh performance liquid chromatography–tandem mass spectrometry (UHPLC-MS/MS) enabled the annotation of 45 additional compounds, highlighting the chemical diversity of *M. sordidum*. The biological potential of the extracts and isolated metabolites was also evaluated through antioxidant, cytotoxic, antimicrobial, and antileishmanial assays.

## 2 Results and Discussion

### 2.1 Chemical Study

A crude ethanol extract (MSE) of the aerial parts of *M. sordidum* was subjected to partition into *n*-hexane, dichloromethane, and ethyl acetate to afford the *n*-hexane (MSH), dichloromethane (MSD), ethyl acetate (MSA), and hydromethanolic (MSHM) fractions, respectively. Successive purification of these fractions by column chromatography led to the isolation of eight compounds (Figure 1), including two new compounds, a sesquiterpene (**5**) and a phenolic glycoside (**8**). The structures of the compounds were assigned based on NMR (^1^H and ^13^C NMR, ^1^H–^13^C HSQC, ^1^H–^13^C HMBC) and HR-ESI-MS data as well as comparison with data reported in the literature. The known compounds were identified as the pentacyclic triterpenes simiarenol (**1**)^15^ and oleanolic acid (**2**)^16^ and the flavonoids hispidulin (**3**),^17^ genkwanin (**6**),^18^ and apigenin (**7**).^19^ Compound **4** was identified as tyrosol^18^ (Figures S1–S6).

**Figure 1:**
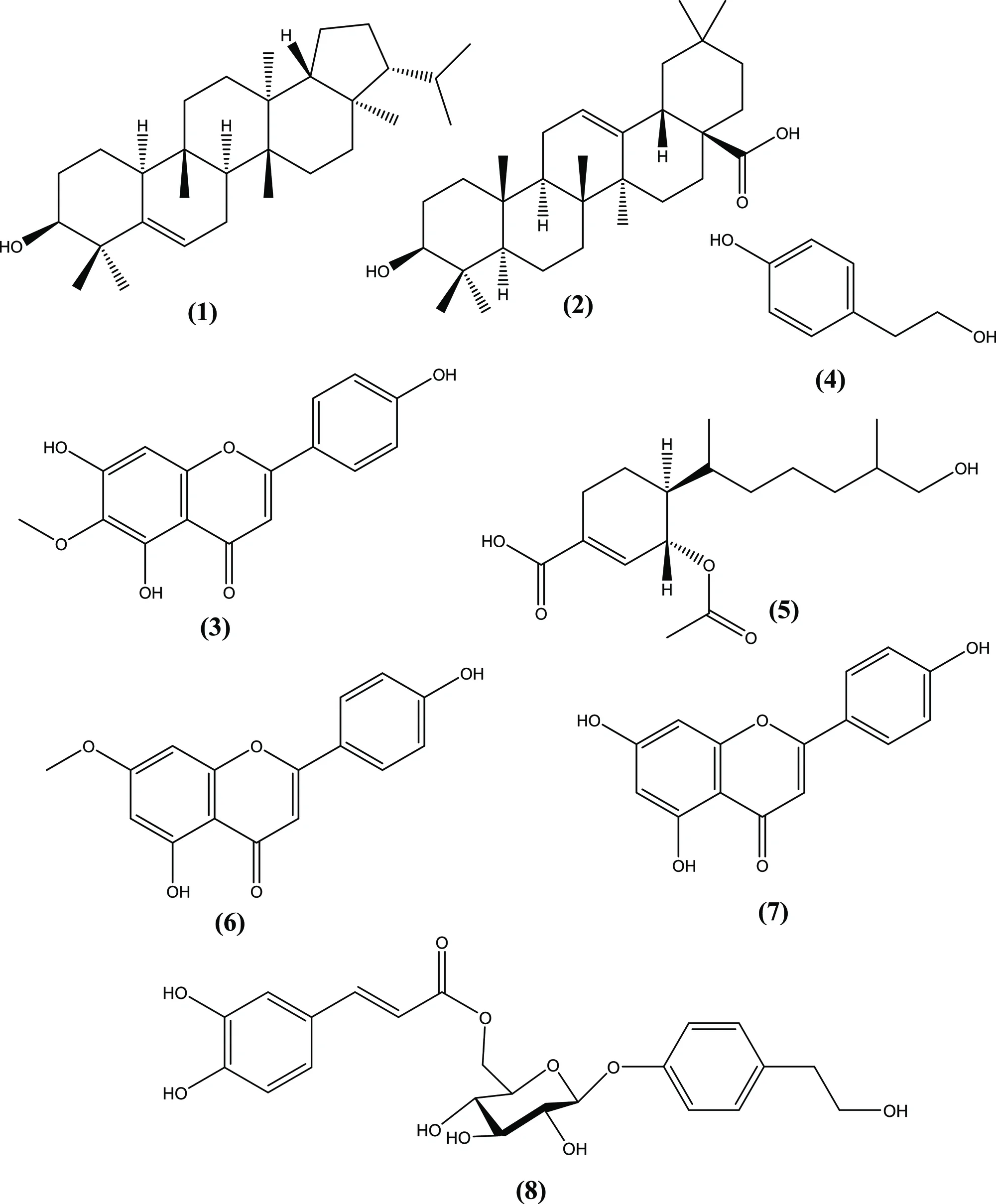
Chemical structures of compounds **1**–**8** isolated from *M. sordidum*.

### 2.2 Structural Elucidation of Bisabolene Derivative

Compound **5** was isolated from the dichloromethane fraction (MSD fraction) as an oil. The molecular formula was established as C_17_H_28_O_5_ based on HRESITOFMS (*m*/*z* 311.1846 [M – H]^−^, calcd 311.1864, error: 5.8). The ^1^H NMR data (Figure S7) for compound **5** are summarized in Table 1 and showed a characteristic olefinic proton signal at δ_H_ 6.77 (H-2, brs). Two methyl groups at δ_H_ 0.78 (H-14; d; *J* = 6.8 Hz) and δ_H_ 0.89 (H-13; d; *J* = 6.7 Hz) were also observed. Signals corresponding to an oxygenated methine and methylene group appeared at δ_H_ 5.43 (H-1; d; *J* = 8.9 Hz) and at δ_H_ 3.49 (H-12a; dd; *J* = 5.8 and 10.5 Hz) and δ_H_ 3.41 (H-12b; dd; *J* = 6.7 and 10.5 Hz), respectively.

**Table 1:** ^1^H (500 MHz) and ^13^C (125 MHz) NMR Data of Sordidic Acid (**5**) in CDCl_3_ (δ in ppm, *J* in Hz)

Sordidic acid **(5)**		
**No**	**dc**	**d_H_**
**1**	70.8	5.43 (1H, d, *J* = 8.9)
**2**	139.0	6.77 (1H, brs)
**3**	132.8	-
**4**	24.1	2.22 (1H, m)
		2.43 (1H, m)
**5**	20.4	1.78 (2H, m)
**6**	41.7	1.67 (1H, m)
**7**	31.3	1.57 (1H, m)
**8**	34.9	2.28 (2H, m)
**9**	24.8	1.35 (1H, m)
		2.47 (1H, m)
**10**	33.1	1.13 (1H, m)
		1.44 (1H, m)
**11**	35.5	1.60 (1H, m)
**12**	68.2	3.49 (1H, dd, *J* = 5.8 and 10.5)
		3.41 (1H, dd, *J* = 6.5 and 10.5)
**13**	16.5	0.89 (3H, d, *J* = 6.7)
**14**	14.3	0.78 (3H, d, *J* = 6.8)
**15**	170.7	-
**1′**	170.9	-
**2′**	21.1	2.12 (3H, s)

The ^13^C NMR data (Figure S8) for compound **5** are summarized in Table 1 and showed 17 carbons signals, including a carboxylic acid group at δ_C_ 170.7 (C-15), two signals corresponding to a double bond at δ_C_ 132.8 (C-3) and δ_C_ 139.0 (C-2), and two methyl groups at δ_C_ 16.5 (C-13) and δ_C_ 14.3 (C-14).

The *g*HMBC spectrum (Figure S9) showed correlations between the signal at δ_H_ 6.77 (H-2) and signals at δ_C_ 170.7 (C-15), 41.7 (C-6), and 24.1 (C-4). A correlation between δ_H_ 3.49 (H-12) and δ_C_ 33.1 (C-10) and 16.5 (C-13) was also observed. The correlations of the signal at δ_H_ 5.43 (H-1) with δ_C_ 41.7 (C-6), δ_C_ 132.8 (C-3), and δ_C_ 170.9 (C-1′) enabled confirmation of the positions of the oxygenated methine and the acetyl group. The main correlations observed in the *g*HMBC spectrum are illustrated in Figure 2. The *g*HSQC (Figure S10) and COSY (Figure S11) data corroborated the proposed structure of compound **5**.

**Figure 2:**
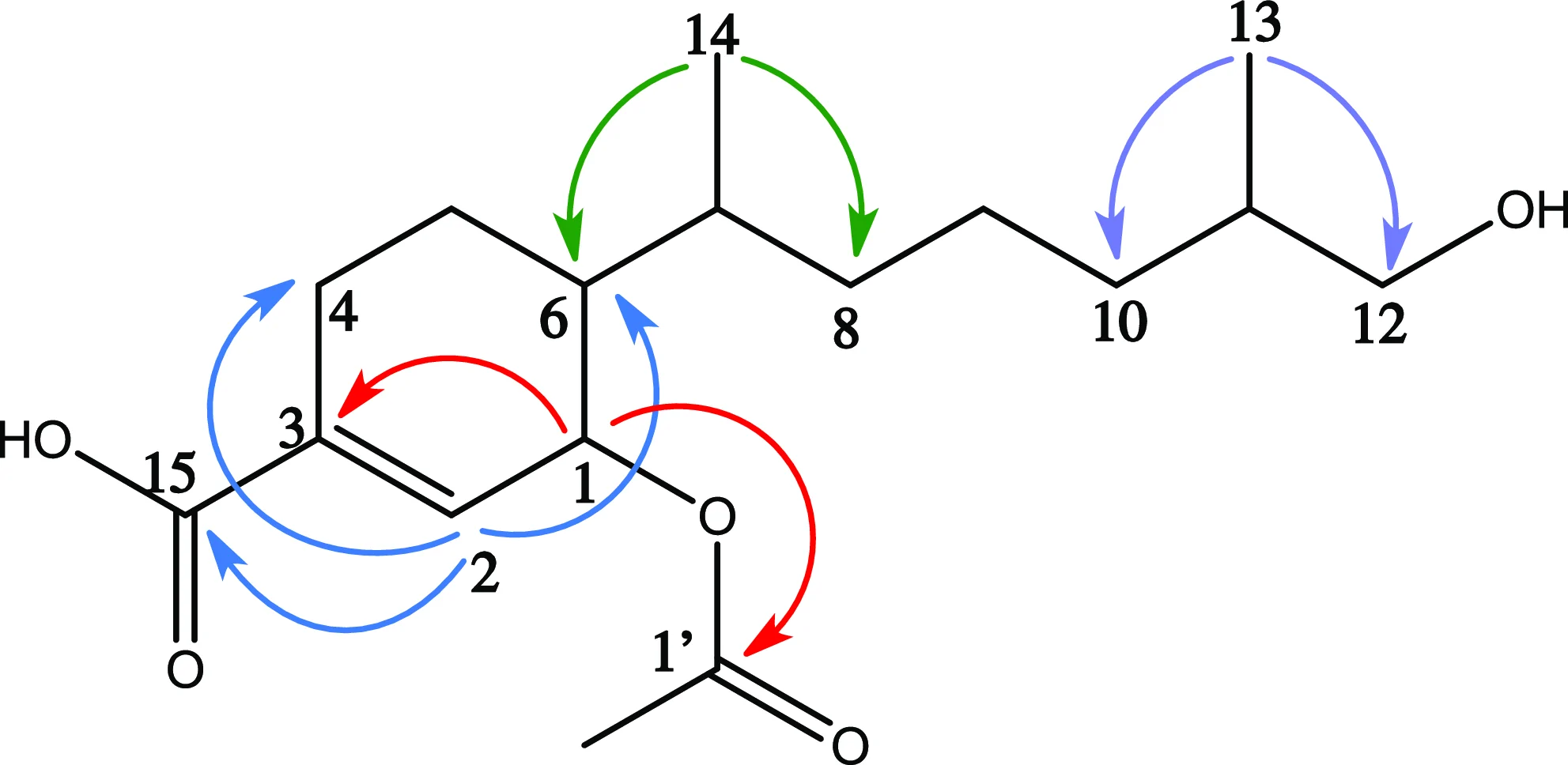
Main correlations observed in the *g*HMBC spectrum of compound **5**.

The *trans* configuration between H-1 and H-2 in compound **5** was established by analysis of the ^3^ *J*_CH_ coupling constants obtained from the pip-HSQMBC-TOCSY-IPAP spectrum (Figure 3). The analysis was carried out using a Bruker Avance III HD spectrometer operating at 500 MHz for ^1^H, with a 5.0 mm probe and the sample diluted in CDCl_3_. The ^3^ *J*_CH_ constants were selected for analysis because their behavior is analogous to that of ^3^ *J*_HH_ with respect to dihedral angles, as described by the Karplus curve. Typically, these constants range from 0 to 9 Hz, with the smallest values observed when the C–C–C-H dihedral angle adopts a *syn* configuration (Figure 4).

**Figure 3:**
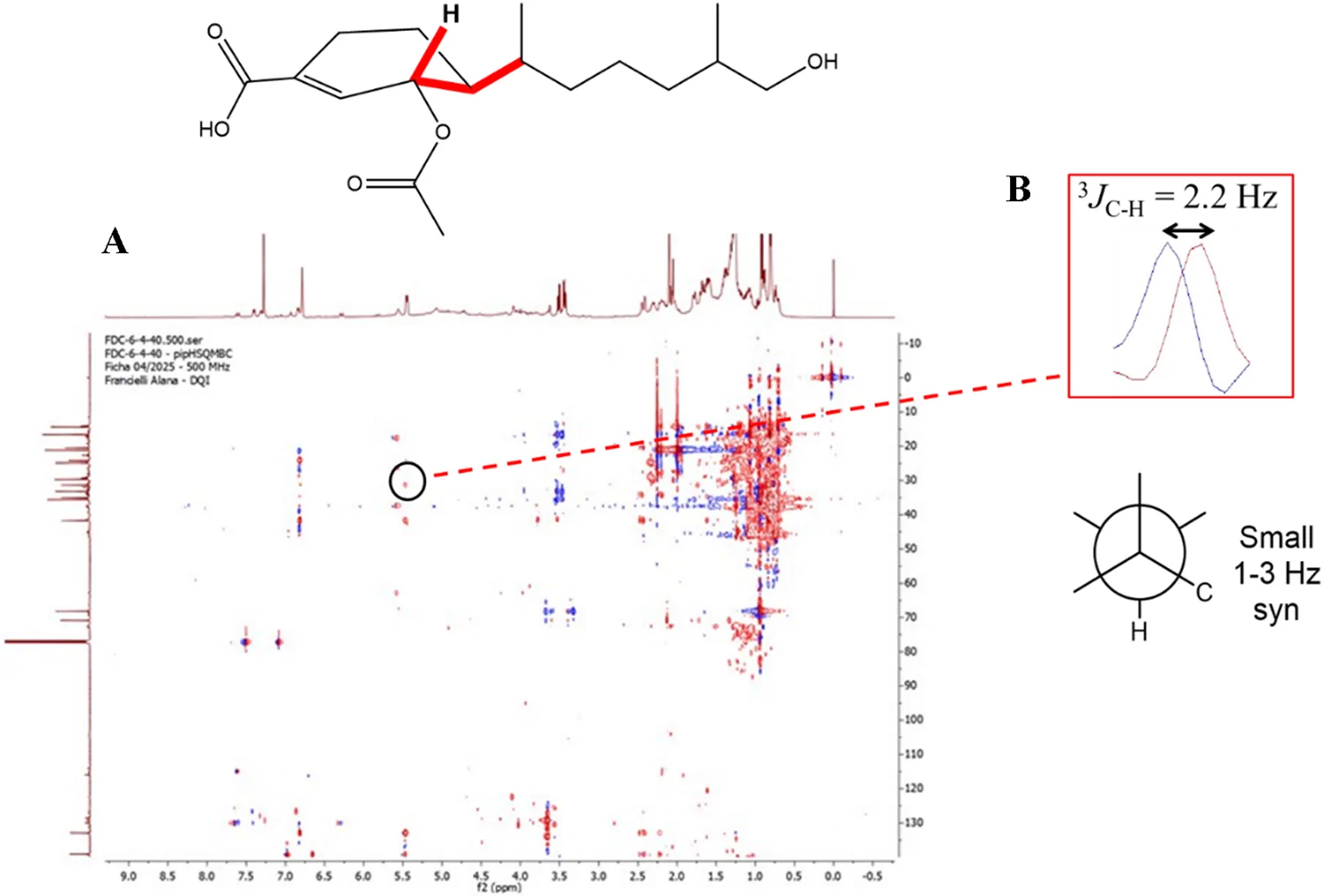
(A) pip-HSQMBC-TOCSY-IPAP spectrum of compound **5**. (B) 1D row slices for H-1 (δ_H_ 5.43) and C-7 (δ_C_ 31.3).

**Figure 4:**
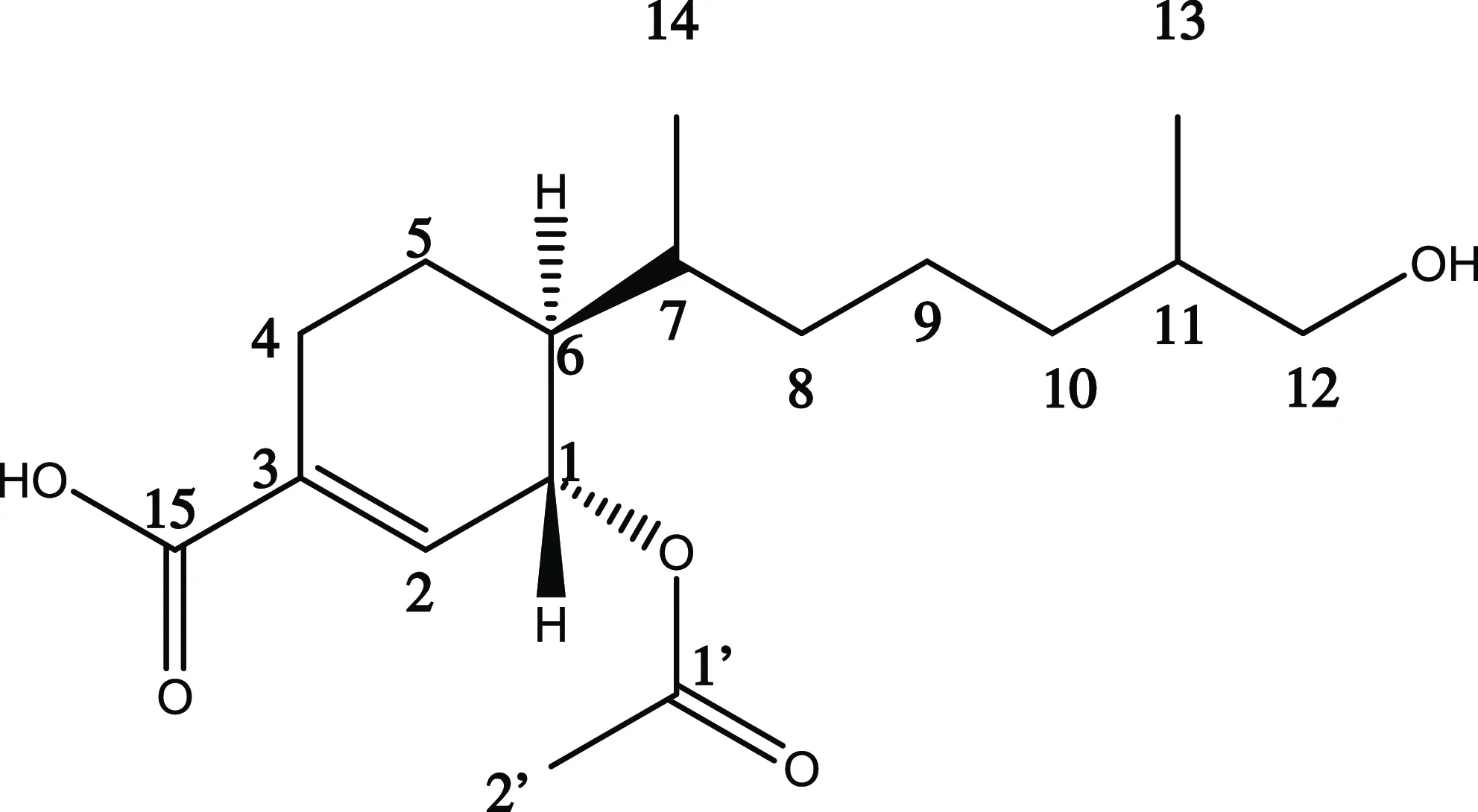
Relative configuration of compound **5** using the pip-HSQMBC-TOCSY-IPAP technique.

The relative configurations of C-7 and C-9 could not be unambiguously assigned. This limitation arises from the high conformational flexibility of the aliphatic side chain, in which free rotation around the C7–C8 and C8–C9 σ-bonds results in a time-averaged set of conformations. Despite this stereochemical ambiguity in the side chain, all 1D and 2D NMR data, combined with HRMS analysis, fully support the proposed structure of compound **5**. Therefore, this previously undescribed sesquiterpene was named sordidic acid.

### 2.3 Structural Elucidation of New Phenolic Glycoside

Compound **8** was isolated from the ethyl acetate fraction (MSA fraction) as a white powder. The molecular formula was established as C_23_H_26_O_10_ based on HRESITOFMS (*m*/*z* 461.1427 [M – H]^−^, calcd 461.1453, error: 5.6). The ^1^H and ^13^C NMR data (Figures S12 and S13) for compound **8** are summarized in Table 2. The ^1^H spectrum showed two *trans-*olefinic protons at δ_H_ 7.47 (H-7′; d; *J* = 15.9 Hz) and δ_H_ 6.26 (H-8′; d; *J* = 15.9 Hz), as well as aromatic proton signals at δ_H_ 7.06 (H-2′; d; *J* = 2.1 Hz), δ_H_ 6.78 (H-5′; d; *J* = 8.1 Hz), and δ_H_ 6.98 (H-6′; dd; *J* = 8.1 and 2.1 Hz), suggesting the presence of a caffeoyl moiety. The ^13^C and *g*HSQC spectrum confirmed the presence of this moiety, with carbon resonances at δ_C_ 126.1 (C-1′), 114.5 (C-2′), 145.9 (C-3′), 146.3 (C-4′), 115.4 (C-5′), 122.0 (C-6′), 149.1 (C-7′), 115.5 (C-8′) and 167.0 (C-9′) (Figure S14).

**Table 2:** ^1^H (300 MHz) and ^13^C (75 MHz) NMR Data of Compounds **8** in DMSO-*d*_6_ (δ in ppm, *J* in Hz)

**8**		
**No**	**dc**	**d_H_**
**1**	101.0	4.84 (1H, d, *J* = 7.3)
**2**	74.4	3.20–3.28^a^
**3**	77.0	3.20–3.28^a^
**4**	70.6	3.20–3.28^a^
**5**	73.8	3.64 (m)^a^
**6a**	64.0	4.42 (1H, dd, *J* = 10.1 and 1.4)
**6b**		4.16 (1H, dd, *J* = 10.1 and 1.4)
**1′**	126.1	-
**2′**	114.5	7.06 (1H, d, *J* = 2.1)
**3′**	145.9	-
**4′**	146.3	-
**5′**	115.4	6.78 (1H, d, *J* = 8.1)
**6′**	122.0	6.98 (1H, dd, *J* = 8.1 and 2.1)
**7′**	149.1	7.47 (1H, d, *J* = 15.9)
**8′**	115.5	6.26 (1H, d, *J* = 15.9)
**9′**	167.0	-
**1”**	156.2	-
**2”**	116.7	6.78 (1H, d, *J* = 8.5)
**3”**	130.3	7.05 (1H, d, *J* = 8.5)
**4”**	133.5	-
**5”**	130.3	7.05 (1H, d, *J* = 8.5)
**6”**	116.7	6.78 (1H, d, *J* = 8.5)
**7”**	38.7	2.60 (2H, t, *J* = 6.9)
**8”**	62.9	3.49 (2H, t, *J* = 6.9)

a The integrations in the sugar region (δ 3.2–3.8 ppm) are affected by water suppression and signal overlap.

Furthermore, the occurrence of two signals integrating for two protons each at δ_H_ 6.78 (H-2″ and H-6″; d; *J* = 8.5 Hz) and δ_H_ 7.05 (H-3″ and H-5″; d; *J* = 8.5 Hz), along with carbon resonances at δ_C_ 156.2 (C-1″), 116.7 (C-2″, C-6″), 130.3 (C-3″, C-5″) and 133.5 (C-4″) indicated the presence of a 1,4-disubstituted phenyl moiety. The glucose moiety was evidenced by the anomeric proton at δ_H_ 4.84 (H-1; d; *J* = 7.3 Hz), which correlated in the *g*HSQC spectrum with the carbon at δ_C_ 101.0 (C-1), as well as by two double doublets at δ_H_ 4.42 (H-6a; dd; *J* = 10.1 and 1.4 Hz) and δ_H_ 4.16 (H-6b; dd; *J* = 10.1 and 1.4 Hz), overlapping signals at δ_H_ 3.20–3.28 (H-2, H-3, and H-4), and a signal at δ_H_ 3.64 (H-5; m).

The connectivity among these three moieties—caffeic acid, tyrosol, and glucose—was established by the *g*HMBC spectrum (Figure S15). Correlations between δ_H_ 4.42 (H-6a) and δ_H_ 4.16 (H-6b) and the carbonyl group of caffeic acid at δ_C_ 167.0 (C-9′) indicated the attachment of C-6 of glucose to the caffeoyl moiety. The attachment of glucose at C-1″ of the phenyl moiety was confirmed by the correlation between the anomeric proton at δ_H_ 4.84 (H-1) and δ_C_ 156.2 (C-1″), supporting C-1″ glycosylation. The main correlations observed in the *g*HMBC and COSY spectra (Figure S16) are illustrated in Figure 5. Thus, the structure of compound **8** was determined to be 1-*O*-tyrosol-6-*O*-caffeoyl-β-d-glucopyranoside.

**Figure 5:**
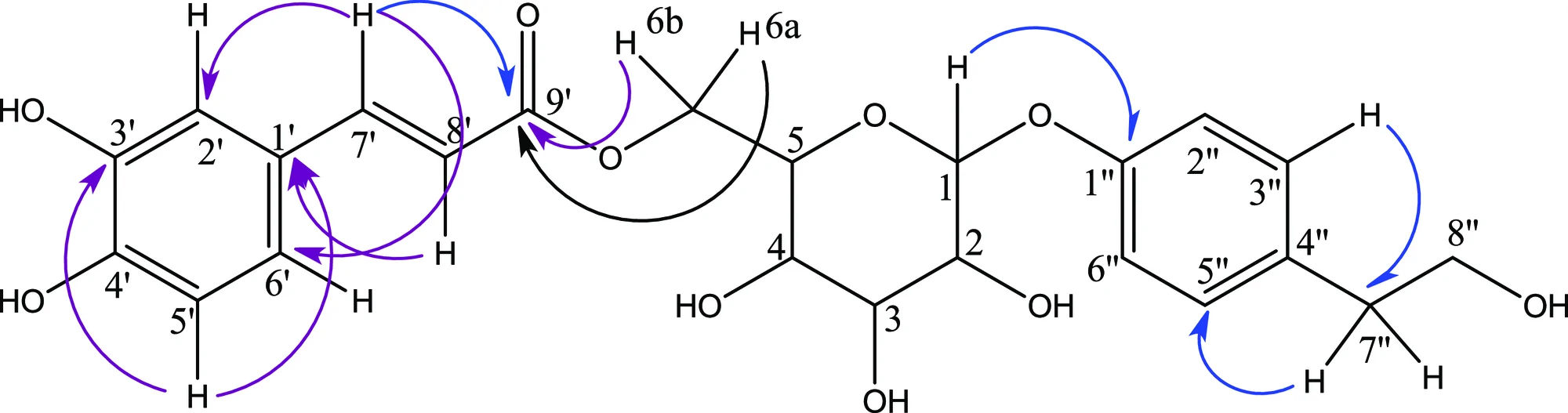
Main correlations observed in the *g*HMBC spectrum of compound **8**.

### 2.4 Dereplication by UHPLC-HRMS/MS

A molecular network was constructed from 317 precursor ions detected in negative ionization mode and visualized as nodes within the network (Figure S17). In total, 45 compounds were putatively identified, including 12 flavonoids, 10 chlorogenic acid derivatives, 5 hydroxycinnamic acid derivatives, 9 terpenes, and 9 miscellaneous compounds. Among the identified flavonoids, hispidulin (**3**; **FLA-05**), genkwanin (**6**; **FLA-04**), and apigenin (**7**, **FLA-06**) were isolated from the aerial parts of *M. sordidum* and were also annotated by the GNPS2 platform, along with seven additional flavonoids (**FLA-01** to **FLA-03**; **FLA-07** to **FLA-10**). One additional flavonoid (**FLA-11**) was putatively identified by manual comparison of its mass spectrum with previously reported literature data (Table 3 and Figure 6).

**Figure 6:**
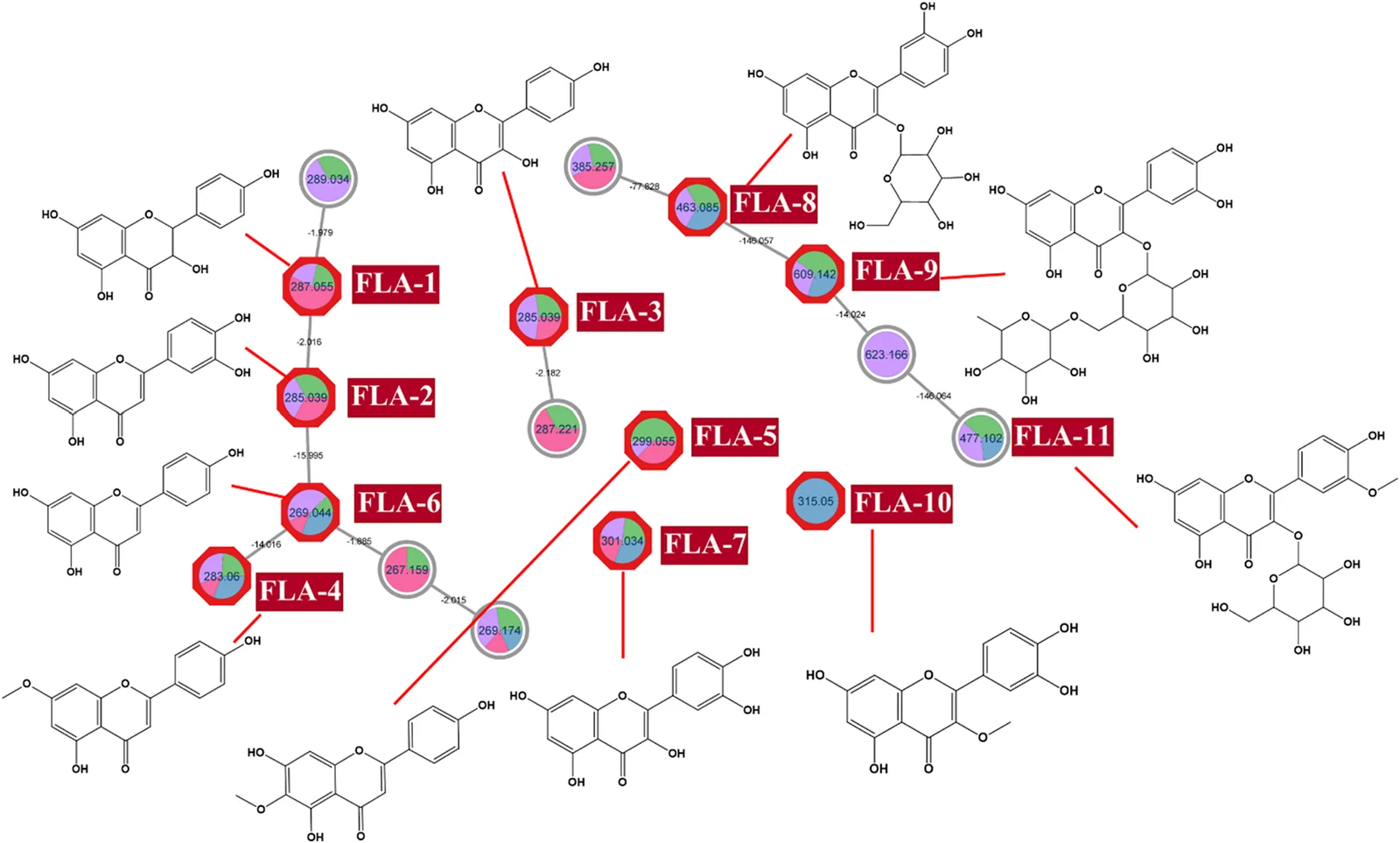
Molecular networking cluster of putatively identified flavonoids in negative ionization mode. Nodes represent detected compounds and are colored according to the respective fraction: green = crude ethanol extract (MSE), purple = ethyl acetate fraction (MSA), pink = dichloromethane fraction (MSD), and blue = hydromethanolic fraction (MSHM).

**Table 3:** Data of the Annotated Compounds in the Fractions of *Moquiniuastrum sordidum* by UHPLC-HRMS/MS and Molecular Networking

Compounds	Molecular formula	Theoretical mass *m*/*z*	Precursor ion *m*/*z*	Mass error/ppm	*t*_R_/min	Main fragment ions	Putative identification
Flavonoids							
**FLA-1**	C_15_H_12_O_6_ [M - H]^−^	287.0561	287.0549	4.2	5.21	259; 177; 151; 125 (b)	Dihydrokaempferol^21^
**FLA-2**	C_15_H_10_O_6_ [M - H]^−^	285.0405	285.0392	4.6	5.39	285 (b); 151; 133	Luteolin^22^
	C_15_H_10_O_6_ [M + H]^+^	287.0550	287.0529	7.3	4.33	287 (b); 153	
**FLA-3**	C_15_H_10_O_6_ [M - H]^−^	285.0405	285.0394	3.8	5.71	285 (b); 255; 229; 185	Kaempferol^21^
	C_15_H_10_O_6_ [M + H]^+^	287.0550	285.0532	6.3	4.57	287 (b); 153	
**FLA-3**(6)	C_16_H_12_O_5_ [M - H]^−^	283.0612	283.0600	4.2	6.38	268 (b); 240; 117	Genkwanin^22,23^
	C_16_H_12_O_5_ [M + H]^+^	285.0757	285.0740	5.9	5.06	270; 242 (b); 167	
**FLA-5**(**3**)	C_16_H_12_O_6_ [M - H]^−^	299.0561	299.0550	3.7	5.70	284 (b); 256; 212; 136	Hispidulin^22^
	C_16_H_12_O_6_ [M + H]^+^	301.0707	301.0685	7.3	4.56	286 (b); 168	
**FLA-6**(**7**)	C_15_H_10_O_5_ [M - H]^−^	269.0455	269.0445	3.7	5.63	269 (b); 151; 117	Apigenin^21^
	C_15_H_10_O_5_ [M + H]^+^	271.0601	271.0589	4.4	4.52	271 (b); 153; 119	
**FLA-7**	C_15_H_10_O_7_ [M - H]^−^	301.0354	301.0341	4.3	5.43	301; 271; 151 (b); 121; 107	Quercetin^21^
	C_15_H_10_O_7_ [M + H]^+^	303.0499	303.0483	5.3	4.39	257; 22; 202; 153; 137; 119; 91 (b)	
**FLA-8**	C_21_H_20_O_12_ [M - H]^−^	463.0882	463.0860	4.7	4.71	300 (b); 271; 255; 161	Hyperoside^22^
**FLA-9**	C_27_H_30_O_16_ [M - H]^−^	609.1461	609.1431	4.9	4.63	300 (b); 271; 161	Rutin^22^
	C_27_H_30_O_16_ [M + H]^+^	611.1607	611.1564	7.0	4.02	303 (b), 163	
**FLA-10**	C_16_H_12_O_7_ [M - H]^−^	315.0510	315.0495	4.8	5.74	300 (b); 271; 255; 243	Isorhamnetin^21,22^
	C_16_H_12_O_7_ [M + H]^+^	317.0656	317.0646	4.6		302 (b); 274; 257	
**FLA-11**	C_22_H_22_O_12_ [M - H]^−^	477.1038	477.1014	5.0	5.11	477; 315; 271; 161 (b)	Isorhamnetin 3-*O*-glucoside^22^
	C_22_H_22_O_12_ [M + H]^+^	479.1184	479.1169	3.1	4.14	317 (b); 163	
**FLA-12**	C_22_H_22_O_12_ [M + H]^+^	315.0863	315.0845	5.7	5.16	300; 272 (b); 168	Pectolinarigenin^24^
Chlorogenic acids derivatives							
**CGA-1**	C_16_H_18_O_9_ [M - H]^−^	353.0878	353.0858	5.7	4.48	191 (b)	Caffeoylquinic acid^22^
	C_16_H_18_O_9_ [M + H]^+^	355.1024	355.1006	5.0	3.99	163 (b); 145; 135; 117; 89	
**CGA-2**	C_31_H_33_O_17_ [M - H]^−^	677.1723	677.1692	4.6	4.67	515; 353; 341; 323; 281; 221; 191 (b); 179; 173; 161; 135	Dicaffeoyl quinic acid hexoside^25^
**CGA-3**	C_16_H_18_O_8_ [M - H]^−^	337.0929	337.0906	6.8	4.68	191 (b); 173; 163	Coumaroyl quinic acid^21^
**CGA-4**	C_25_H_24_O_12_ [M - H]^−^	515.1195	515.1165	3.9	4.92	191 (b); 179; 173; 161; 135	Dicaffeoyl quinic acid^22^
	C_25_H_24_O_12_ [M + H]^+^	517.1341	517.1325	3.1	4.19	163 (b); 145; 117; 89	
**CGA-5**	C_25_H_24_O_11_ [M - H]^−^	499.1246	499.1224	4.4	5.17	337; 191; 179; 173 (b); 163; 155	Coumaroyl-caffeoylquinic acid^26^
	C_25_H_24_O_11_ [M + H]^+^	501.1391	501.1358	6.6	4.29	163 (b); 147; 119; 91	
**CGA-6**	C_26_H_26_O_12_ [M - H]^−^	529.1351	529.1329	4.1	5.22	193; 179; 173 (b); 161; 135	[(3,4-dihydroxy phenyl)prop-2-enoyl]oxy-1,5-dihydroxy-4-[3-(3-hydroxy-4-methoxy phenyl) prop-2-enoyl] oxycyclohexane-1-carboxylic acid^22^
	C_26_H_26_O_12_ [M + H]^+^	531.1497	531.1478		4.28	177; 163 (b); 145; 89	
**CGA-7**	C_34_H_30_O_15_ [M - H]^−^	677.1512	677.1481	4.6	5.25	515; 353; 335; 291; 179; 173 (b); 135	3,4,5-tri-*O*-caffeoyl quinic acid^22^
	C_34_H_30_O_15_ [M + H]^+^	679.1657	679.1616	6.0	4.30	163 (b); 145; 117; 89	
**CGA-8**	C_17_H_20_O_9_ [M - H]^−^	367.1035	367.1007	7.6	4.72	191; 179; 161; 135 (b)	Methyl chlorogenate^22^
**CGA-9**	C_34_H_32_O_15_ [M - H]^−^	691.1668	691.1641	3.9	5.47	529; 367; 335; 193; 179; 173; 161 (b); 135	Dicaffeoyl feruloyl quinic acid^27^
**CGA-10**	C_35_H_35_O_15_ [M - H]^−^	705.1825	705.1774	7.2	5.61	543; 381; 363; 269; 179; 161 (b); 135	Diferuloyl caffeoyl quinic acid^27^
Hydroxycinnamic acids derivatives							
**Caffeic acid**	C_9_H_8_O_4_ [M + H]^+^	181.0495	181.0478	9.4	4.10	163; 135; 117; 89 (b)	Caffeic acid^21^
**CAH-1**	C_15_H_18_O_9_ [M - H]^−^	341.0878	341.0853	6.7	4.34	281; 221; 179; 161 (b); 135	Caffeoyl glucopyranose^27^
**CAH-2**	C_29_H_36_O_15_ [M - H]^−^	623.1981	623.1972	3.2	4.57	623; 461; 323; 161(b)	Isoacteoside^28^
**CAH-3**	C_24_H_24_O_12_ [M - H]^−^	503.1195	503.1179	3.2	4.76	323; 221; 179 (b); 161; 135	Dicaffeoyl glucopyranose^27^
**CAH-4**(8)	C_23_H_26_O_10_ [M - H]^−^	461.1453	461.1427	5.6	4.80	323; 179; 163 (b); 137	1-*O*-tyrosol-6-*O*-caffeoyl-β-d-glucopyranoside (Figure 8)
**CAH-5**	C_23_H_24_O_8_ [M - H]^−^	445.1504	445.1478	5.8	4.95	163; 145 (b); 137; 117	1-*O*-tyrosol-6-*O*-coumaroyl-β-d-glucopyranoside (Figure 9)
Terpenes							
**TER-01**(5)	C_17_H_28_O_5_ [M + H]^+^	311.1864	311.1846	5.8	6.10	271; 251 (b); 223; 203; 177; 121	Sordidic acid
	C_17_H_28_O_5_ [M + H - H_2_O]^+^	253.1798	253.1785	5.1	4.83	147; 133; 105 (b); 91; 79	
**TER-02**	C_20_H_34_O_6_ [M - H + HCOOH]^−^	399.2388	399.2372	4.0	5.31	353 (b); 307; 259	5-[5-hydroxy-3-(hydroxymethyl)pentyl]-8a-(hydroxymethyl)-5,6-dimethyl-3,4,4a,6,7,8-hexahydronaphthalene-1-carboxylic acid^22^
**TER-03**	C_20_H_30_O_4_ [M - H + HCOOH]^−^	379.2126	379.2103	5.3	6.80	333 (b); 289; 259	1,10a-dihydroxy-4,4,7,11b-tetra methyl-1,2,3,4a, 5,6,6a,7,11,11a-decahydronaphtho[2,1-*f*][1]benzofuran-9-one^22^
**TER-04**	C_30_H_46_O_3_ [M + H]^+^	455.3520	455.3494	5.7	6.77	201, 187; 133; 119; 107; 95 (b); 81	3-Hydroxy-11-ursen-28,13-olide^22^
**TER-05**(2)	C_30_H_46_O_2_ [M + H–H_2_O]^+^	439.3571	439.3554	3.8	9.53	393; 213; 137; 109; 95 (b); 81	Oleanolic acid^29^
**TER-06**	C_30_H_50_O_2_ [M + H]^+^	443.3884	443.3858	5.8	11.06	319; 217; 203; 163; 135; 109 (b); 95; 79	Uvaol^30^
**TER-07**	C_13_H_20_O_2_ [M + H]^+^	209.1536	209.1528	3.8	3.94	133; 121; 107; 91 (b); 80	1*H*-Indene-3-carboxylic acid, 3a,4,5,6,7,7a-hexahydro-3a,7,7-trimethyl-^22^
**TER-08**	C_30_H_50_O_2_ [M + H]^+^	443.3884	443.3855	6.5	11.03	269; 191; 147; 121; 109; 107; 95 (b); 81	Betullin^22^
**TER-09**	C_30_H_48_O_3_ [M + H]^+^	439.3571					Betulinic Acid^22^
Miscellaneous							
**M-01**	C_12_H_22_O_11_ [M + H–H_2_O]^+^	325.1129	325.1112	5.2	0.86	163; 145; 127; 97;	Turanose^22^
						85 (b)	
	C_12_H_22_O_11_ [M - H]^−^	341.1089	341.1074	1.3	0.90	191; 161; 136 (b); 113; 101; 85	
**M-02**	C_6_H_10_O_7_ [M - H]^−^	195.0510	195.0500	5.1	0.88	195 (b); 129; 99; 87; 75	Gluconic acid^21^
**M-03**	C_22_H_36_O_4_ [M + H]^+^	365.2686	365.2664	6.0	6.60	159; 119 (b); 105; 95; 81	[12-hydroxy-6-(hydroxymethyl)-10-methyl-2-(4-methylpent-3-enyl)dodeca-2,6,10-trienyl] acetate^21^
**M-04**	C_35_H_36_N_4_O_5_ [M + H]^+^	593.2758	593.2731	4.5	11.58	593 (b); 533; 505; 460	Pheophorbide A^31^
**M-05**	C_36_H_38_N_4_O_5_ [M + H]^+^	607.2915	609.2875	6.6	13.84	607 (b); 547; 505; 461	Pheophorbide A methyl ester^31^
**M-06**	C_11_H_16_O_3_ [M + H]^+^	197.1172	197.1162	5.1	4.34	179; 161; 133; 105 (b); 91; 77	2(4*H*)-Benzofuranone, 5,6,7,7a-tetrahydro-6-hydroxy-4,4,7a-trimethyl-, (6*S*,7a*R*)-^22^
**M-07**	C_24_H_32_O_7_ [M + H–H_2_O]^+^	415.2115	415.2097	4.3	5.42	221; 187; 135; 119 (b); 107; 95; 91	Gelomulide N^22^
**M-08**		329.2333	329.2319	4.2		331; 299; 271; 229; 211 (b); 171; 139;99	FA 18:1 + 3O^21^
**M-09**	C_17_H_17_NO_3_ [M - H]^−^	282.1281	282.1119	6.0	5.24	239; 216; 162; 132; 119 (b)	4-coumaroyl-tyramine^22^
	C_17_H_17_NO_3_ [M + H]^+^	284.1281	284.1267	4.9	4.29	147 (b); 121; 103; 91; 77	

Chlorogenic acid derivatives (CGAs) sharing the same carbon skeleton typically generate similar fragmentation patterns in mass spectrometry, with diagnostic fragment ions that can be used for the screening and characterization of CGA analogues. In negative ionization mode, the characteristic fragments include *m*/*z* 191.056 and *m*/*z* 173.045, both originating from the quinic acid moiety, as well as *m*/*z* 179.034 and *m*/*z* 135.045, derived from the caffeic acid moiety.^20^

Based on these consistent diagnostic fragments, application of the GNPS2MS2 *m*/*z* highlight tool enabled the identification of nodes exhibiting these diagnostic ions within the molecular network. As a result, 30 nodes were highlighted, of which seven were putatively annotated by the GNPS platform and three additional nodes were putatively identified by manual comparison of their mass spectra with data reported in the literature (**CGA-01** to **CGA-10**) (Table 3 and Figure 7).

**Figure 7:**
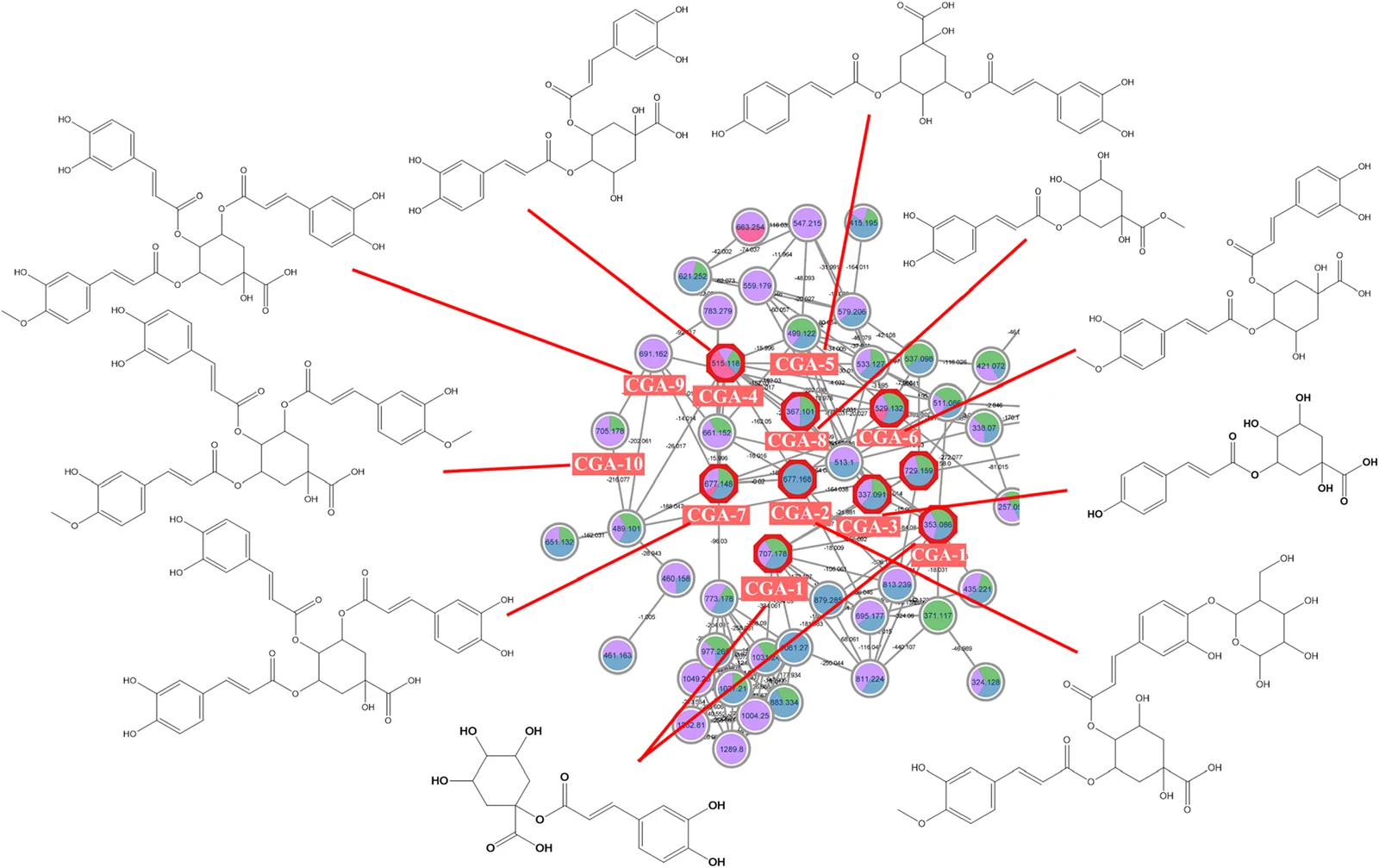
Molecular networking cluster highlighting putative chlorogenic acid derivatives based on MS/MS data in the negative ionization mode. Nodes represent detected compounds and are colored according to the respective fraction: green = crude ethanol extract (MSE), purple = ethyl acetate fraction (MSA), pink = dichloromethane fraction (MSD), and blue = hydromethanolic fraction (MSHM).

A caffeic acid hexoside (**CAH-01**) was annotated by the GNPS platform. The observed MS/MS fragmentation pattern was consistent caffeic acid derivatives, displaying diagnostic product ions at *m*/*z* 179.033 and 135.043, as well as an ion at *m*/*z* 161.023 corresponding to H_2_O loss from the fragment at *m*/*z* 179.033.^20^ Similar fragmentation behavior was observed for compound **8** (**CAH-04**), which is reported here for the first time. In addition to the characteristic caffeic acid product ions, compound **8** yielded an ion at *m*/*z* 137.059, consistent with a tyrosol moiety (Table 3 and Figure 8), supporting the presence of a tyrosol unit in the structure.

**Figure 8:**
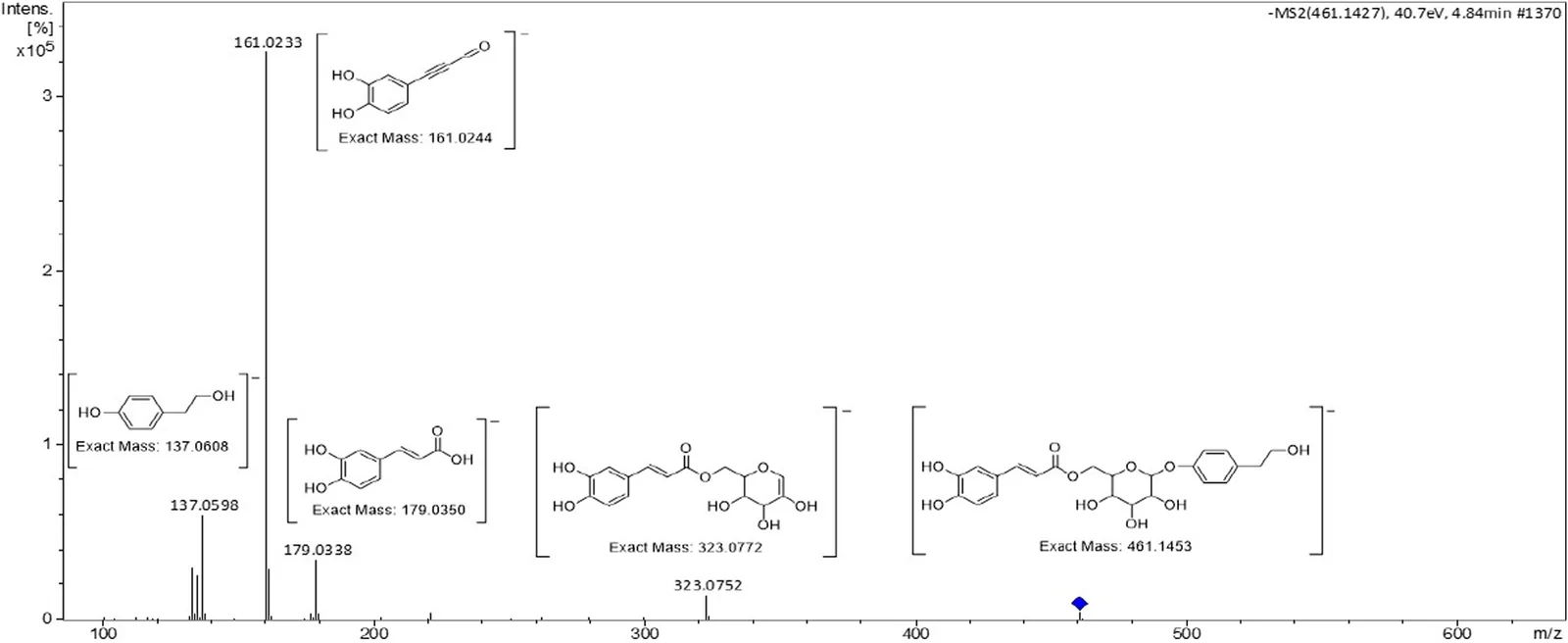
MS/MS fragmentation pattern of compound **8** (**CAH-04**) with diagnostic ions supporting caffeic acid and tyrosol moieties in negative ionization mode.

Based on the MS/MS fragmentation pattern in Figure 8, we inferred a related structure (**CAH-05**) bearing a coumaroyl group instead of a caffeoyl group (Table 3 and Figure 9). This assignment is supported by diagnostic ions consistent with coumaric acid derivatives (e.g., [coumaric acid - H]^−^ at *m*/*z* 163).

**Figure 9:**
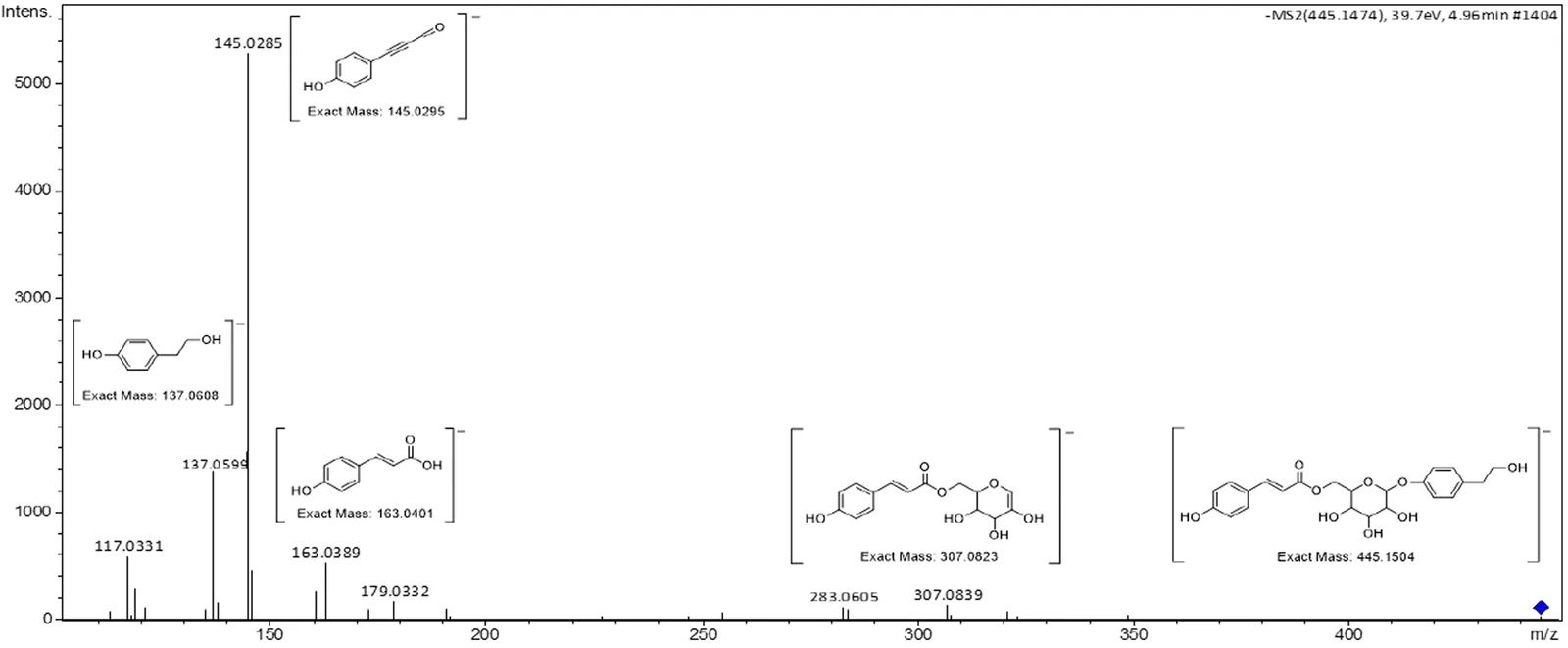
MS/MS fragmentation pattern of compound **CAH-05** consistent with a coumaroyl moiety.

### 2.5 Antioxidant Assay

The antioxidant activity of the crude extract, fractions, and isolated compounds was evaluated using the ABTS radical scavenging and ferric reducing antioxidant power (FRAP) assays (Table 4). Overall, solvent partitioning markedly influenced the antioxidant profile of *M. sordidum*. While the crude extract (MSE) exhibited considerable antioxidant activity (ABTS: 1215.6 μmol Trolox g^–1^; FRAP: 4734.4 μmol Fe­(II) g^–1^), this activity was concentrated in the ethyl acetate fraction (MSA), which displayed the highest values among all samples (ABTS: 2763.0 μmol Trolox g^–1^; FRAP: 9652.6 μmol Fe­(II) g^–1^). By contrast, the dichloromethane fraction (MSD) exhibited substantially lower antioxidant activity (ABTS: 235.6 μmol Trolox g^–1^; FRAP: 711.8 μmol Fe­(II) g^–1^), indicating that the antioxidant compounds were preferentially enriched in the more polar fraction. This behavior is consistent with the higher affinity of phenolic compounds, particularly flavonoids and phenolic glycosides, for solvents of intermediate-to-high polarity such as ethyl acetate.

**Table 4:** Antioxidant Activity of the Crude Extract and Fractions from *Moquiniastrum sordidum* Aerial Parts Determined by ABTS and FRAP Assays^a^

Samples	ABTS (μmol Trolox g^–1^)	FRAP (μmol de Fe(II) g^–1^)
MSE	1215.6	4734.4
MSH	nd	nd
MSD	235.6	711.8
MSA	2763.0	9652.6
Simiarenol **(1)**	<10	75.9
Hispidulin (**3**) and Tyrosol (**4**)	64.0	480.7
Genkwanin (**6**)	<10	123.3
Apigenin (**7**)	1440.4	3325.1
New phenolic (**8**)	1111.1	4293.0

a MSE (crude extract); MSH (hexanic fraction); MSD (dichloromethane fraction); MSA (ethyl acetate fraction); nd = not determined.

Among the isolated compounds, apigenin (**7**) exhibited the highest antioxidant activity, with values of 1440.4 μmol Trolox g^–1^ in the ABTS assay and 3325.1 μmol Fe­(II) g^–1^ in the FRAP assay. By contrast, hispidulin (**3**) and tyrosol (**4**) showed only moderate activity, whereas genkwanin (**6**) and simiarenol (**1**) were essentially inactive in the ABTS assay (<10 μmol Trolox g^–1^). These findings demonstrate that relatively small structural modifications substantially influence the antioxidant capacity of flavonoids.

The superior antioxidant activity of apigenin (**7**) is consistent with previous structure–activity relationship studies demonstrating that free phenolic hydroxyl groups play a central role in radical scavenging and reducing capacity. In particular, the presence of an unsubstituted 4′-hydroxyl group enhances hydrogen atom donation and electron transfer, whereas methoxylation, as observed in genkwanin (7-OCH_3_) and hispidulin (6-OCH_3_), generally reduces antioxidant activity by decreasing the number of free phenolic hydroxyl groups available for these mechanisms.^32,33^

The new phenolic glycoside derivative (**8**) also exhibited pronounced antioxidant activity in both assays (ABTS: 1111.1 μmol Trolox g^–1^; FRAP: 4293.0 μmol Fe­(II) g^–1^), ranking among the most active compounds isolated in this study. This result is in agreement with previous reports describing strong antioxidant activity for structurally related caffeoylated phenolic glycosides, such as (*E*)-4-hydroxymethylphenyl-6-*O*-caffeoyl-β-d-glucopyranoside isolated from *Lagenaria siceraria*, in which the caffeoyl moiety plays a major role in radical scavenging and reducing capacity owing to its catechol functionality.^34^ The high antioxidant activity observed for compound **8** further supports the contribution of phenolic constituents to the marked antioxidant capacity of the ethyl acetate fraction.

Although compound **5** (sordidic acid) was not individually evaluated in the antioxidant assays, it was isolated from the dichloromethane fraction, which exhibited relatively low antioxidant activity in both the ABTS and FRAP assays. Therefore, the antioxidant contribution of compound **5** cannot be established based on the present data and should be addressed in future studies using the purified compound.

### 2.6 *In Vitro* Antiproliferative Assay

The antiproliferative activity was evaluated using the MTT assay. For an initial screening of cytotoxic potential, the crude extract of *M. sordidum* and its fractions were assayed against HCT-116 colorectal carcinoma cells at two concentrations (50 and 5 μg mL^–1^). Preliminary cytotoxic screening showed that the dichloromethane fraction (MSD) inhibited proliferation by 92% at 50 μg mL^–1^, while activity dropped to 0% at 5 μg mL^–1^ (Table S1).

*M. polymorphum* is the most extensively studied related species with regard to the presence of bioactive compounds, largely because of its use in Brazilian folk medicine as an auxiliary treatment for inflammation and other debilitating diseases.^13^ Interestingly, the cytotoxic activity of extracts from specimens of this species has been associated with their dichloromethane fractions.^35–37^ Investigation of the cytotoxic potential of the ethanolic extract of *M. polymorphum* subsp. *polymorphum* in activated hepatic stellate cells demonstrated that the crude extract and dichloromethane:ethanol fraction (90:10, v/v) inhibited the expression of several cytokines related to the cell cycle, in addition to reducing cell proliferation by inducing a quiescent state.^35^

Other studies have further investigated the anticancer potential of compounds isolated from the dichloromethane fraction of *M. polymorphum* subsp. *floccosum*. Bioguided fractionation of the crude extract using 10 cancer cell lines led to the isolation of six compounds and the identification of the dimeric lactones 10-deoxygochnatiolide A and gochnatiolide A, which exhibited potent cytotoxic activity.^36^ Later, researchers investigated the *in vivo* antineoplastic properties of the dichloromethane fraction of subsp. *floccosum*, which is rich in sesquiterpene lactones, and found that male Wistar rats treated with this fraction showed tumor reductions of up to 74%. According to these investigations, this effect is due to inhibition of the TNF-α signaling pathway and induction of apoptosis through p53 activation and Bax gene expression.^37^

In this work, we demonstrated that although the crude extract of *M. sordidum* did not show significant cytotoxic activity in HCT-116 carcinoma cells, the dichloromethane fraction (MSD) inhibited cell growth by more than 90%, and the majority of the identified isolated compounds were obtained from this fraction.

### 2.7 Antimicrobial Assay

The crude extract and fractions of *M. sordidum* were tested against four pathogenic bacterial species: *Staphylococcus aureus* (ATCC 25923), *B. cereus* (ATCC 11778), *Salmonella enterica* subsp. *enterica* serovar Enteritidis (ATCC 13076), and *Escherichia coli* (ATCC 25922). Oleanolic acid (**2**) and apigenin (**7**) were active against the Gram-positive bacterium *B. cereus*, with MIC values of 130 and 30 μg mL^–1^, respectively. The remaining fractions and crude extract showed no inhibition of the bacteria at the highest concentration tested (500 μg mL^–1^).

### 2.8 Antileishmanial Assay

The antileishmanial activity of the crude extract (MSE), its fractions (MSD, MSA, and MSHM), and the new sesquiterpene sordidic acid (**5**) was evaluated against promastigote and amastigote forms of *Leishmania amazonensis*, in parallel with their cytotoxicity toward J774A.1 macrophages (Table 5). Overall, fractionation significantly improved both potency and selectivity, particularly against the clinically relevant intracellular amastigote stage.

**Table 5:** Evaluation of the Antileishmanial Activity and Cytotoxicity of Extracts and Fractions of *Moquiniastrum sordidum* ^a^

		*Leishmania amazonenses*			
	J774A.1	Promastigotes		Amastigotes	
Sample	CC_50_ ± SD (μg mL^–1^)	IC_50_ ± SD (μg mL^–1^)	SI	IC_50_ ± SD (μg mL^–1^)	SI
MSE	85.02 ± 4.85	99.87 ± 7.02	0.85	13.36 ± 1.67	6.36
MSD	64.77 ± 7.39	52.15 ± 2.51	1.24	8.23 ± 0.97	7.87
MSA	861.73 ± 26.30	>200	nd	nt	nd
MSHM	>1000	>200	nd	nt	nd
Sordidic acid (**5**)	36.69 ± 2.17	122.54 ± 11.34	0.30	12.84 ± 0.74	2.86
Miltefosine	24.45 ± 3.43	9.49 ± 0.31	2.58	0.89 ± 0.08	27.47

a MSE (crude extract); MSD (dichloromethane fraction); MSA (ethyl acetate fraction); MSHM (hydromethanolic fraction); IC_50_ = inhibitory concentration for 50% of protozoa. CC_50_ = cytotoxic concentration for 50% of cells. SD = standard deviation. SI = selectivity index (CC_50_/IC_50_). nd = not determined. nt = not tested.

MSE exhibited moderate activity against promastigotes (IC_50_ = 99.87 μg mL^–1^) but demonstrated a significantly more potent effect against intracellular amastigotes (IC_50_ = 13.36 μg mL^–1^), resulting in a selectivity index (SI) of 6.36. This preferential activity toward the amastigote stage is pharmacologically relevant because it represents the clinically relevant replicative form within the mammalian host and the primary target for antileishmanial chemotherapy.

The results revealed a significant increase in activity following fractionation, with the dichloromethane fraction (MSD) displaying improved potency against both promastigotes (IC_50_ = 52.15 μg mL^–1^) and amastigotes (IC_50_ = 8.23 μg mL^–1^), along with an increased SI (7.87). By contrast, the ethyl acetate (MSA) and hydromethanolic (MSHM) fractions were inactive (IC_50_ > 200 μg mL^–1^), indicating that the bioactive constituents were preferentially concentrated in the MSD fraction. This behavior is consistent with the extraction profile of moderately lipophilic metabolites, such as terpenoids and methoxylated flavonoids, which are commonly associated with antileishmanial effects.

The new sesquiterpene, sordidic acid (**5**), isolated from fraction MSD, retained activity against amastigotes (IC_50_ = 12.84 μg mL^–1^) but showed reduced selectivity (SI = 2.86), suggesting that the higher activity of MSD may result from combined or synergistic effects among multiple constituents rather than a single active compound.

Cytotoxicity analysis revealed CC_50_ values of 85.02 μg mL^–1^ (MSE) and 64.77 μg mL^–1^ (MSD), indicating moderate toxicity toward host cells. Nevertheless, the SI value obtained for MSD, particularly against amastigotes, supports a selective antiparasitic effect within this fraction.

Miltefosine, used as the reference drug, exhibited significantly higher potency (IC_50_ = 0.89 μg mL^–1^ for amastigotes) and selectivity (SI = 27.47). Nevertheless, the activity demonstrated by the MSD fraction remains noteworthy for a plant-derived fraction, especially considering the complexity of the extract and the absence of compound-level optimization.

The antileishmanial activity observed herein is consistent with previous reports on Asteraceae species, particularly those belonging to the former genus *Gochnatia*. Sesquiterpene lactones and related terpenoids isolated from *M. polymorphum* have been associated with pronounced biological activities, including cytotoxic and antiparasitic effects.^6,36^ In addition, flavonoids such as apigenin, identified in the present study, have been reported to exhibit antileishmanial activity, potentially through mechanisms involving mitochondrial dysfunction and the induction of oxidative stress in the parasite.^38^

Taken together, these results demonstrate that systematic fractionation significantly enhances the antileishmanial activity of *M. sordidum*, with the dichloromethane fraction (MSD) emerging as the most promising sample. The data also suggest that the observed activity may arise from synergistic or additive effects among the metabolites enriched in this fraction.

## 3 Experimental Section

### 3.1 General Experimental Procedures

Chromatographic separations were performed on silica gel (60–230 mesh), flash silica gel (230–400 mesh), and Sephadex LH-20 (Sigma, USA). Thin-layer chromatography (TLC) was performed on normal-phase precoated silica gel 60G or 60GF254 plates (Merck, Germany). Compounds on TLC plates were visualized by UV irradiation at 254 and 366 nm and/or by spraying with H_2_SO_4_:anisaldehyde:acetic acid (1:0.5:50 mL) solution followed by heating at 100 °C. Nuclear magnetic resonance (NMR) spectra were recorded on a Bruker Avance Neo 300 NanoBay spectrometer (Germany) operating at 300 MHz for ^1^H NMR and 75.5 MHz for ^13^C NMR and at 500 MHz for ^1^H NMR and 125 MHz for ^13^C NMR, using deuterated chloroform (CDCl_3_) and dimethyl sulfoxide (DMSO-d6) as solvents. UHPLC analyses were performed using a Shimadzu Nexera X2 instrument (Japan) equipped with a CBM-20A system controller, two LC-30AD pumps, a CTO-30A column oven, and a SIL-30AC autosampler. Mass spectra were recorded on Bruker IMPACT II mass spectrometer (USA) equipped with an electrospray ionization source (ESI) operating in positive and negative ion modes, a quadrupole time-of-flight (Q-TOF) analyzer, and a multichannel plate (MCP) detector.

### 3.2 Plant Material

The aerial parts of *M. sordidum* (Less.) G. Sancho were collected in April 2019 at Parque Estadual Vale do Codó, specifically around Cachoeira do Lago Azul (24°16′34″S, 49°41′36″W; alt. 843 m), in the municipality of Jaguariaíva, Campos Gerais region of Paraná State (southern Brazil), and were identified by Dr. Marta Regina Barrotto do Carmo. A voucher specimen was deposited in the herbarium of Universidade Estadual de Ponta Grossa (HUPG 22446; SISGEN A8C0C1D).

### 3.3 Extraction and Isolation

The dried and powdered plant material (668 g) was subjected to exhaustive maceration with ethanol at room temperature. The solvent was removed by evaporation under vacuum, yielding the crude extract (MSE; 118 g). An aliquot of MSE (79 g) was solubilized in MeOH/H_2_O (1:1, 400 mL, v/v) and partitioned successively with *n*-hexane (4 × 100 mL), dichloromethane (3 × 100 mL), and ethyl acetate (5 × 100 mL). The fractions were concentrated, yielding the hexane (MSH; 11.9 g), dichloromethane (MSD; 28.9 g), ethyl acetate (MSA; 24.8 g), and hydromethanolic (MSHM; 5.8 g) fractions.

The MSH fraction (11.9 g) was subjected to silica gel 60 CC and eluted with a gradient of hexane and EtOAc of increasing polarity, providing subfractions MSH-1 to MSH-14. Subfraction MSH-4 (48.2 mg) yielded compound **1**. A portion of the MSD fraction (10.1 g) was subjected to vacuum liquid chromatography on a silica gel column with a gradient of *n*-hexane–EtOAc to yield subfractions MSD-1 to MSD-14. Subfraction MSD-6 (1.2 g) was subjected to flash silica gel CC and eluted with a gradient of hexane and EtOAc of increasing polarity, providing subfractions MSD-6–1 to MSD-6–15. Subfraction MSD-6–8 (103.1 mg) yielded compound **2**. Subfraction MSD-8 (1.1 g) was subjected to flash silica gel CC and eluted with a gradient of hexane and EtOAc of increasing polarity, providing subfractions MSD-8–1 to MSD-8–35. Subfraction MSD-8–18 (88.9 mg) afforded a mixture of compounds **3** and **4**, and subfraction MSD-8–25 (42.1 mg) afforded compound **5**. Subfractions MSD-12 (3.0 g) and MSD-13 (0.21 g) were subjected to flash silica gel CC using gradient of *n*-hexane and EtOAc of increasing polarity as the eluent to yield subfractions MSD-12–1 to MSD-12–22 and MSD-13–1 to MSD-13–19, respectively. Subfraction MSD-12–8 (92.5 mg) afforded compound **6**, and subfraction MSD-13–7 (32.8 mg) afforded compound **7**. An aliquot of the MSA fraction (20 g) was subjected to Sephadex LH-20 CC using H_2_O and MeOH as eluents in a decreasing-polarity gradient, resulting in subfractions MSA-1 to MSA-9. Compound **8** was obtained from subfraction MSA-4 (258 mg), and compound **7** was reisolated from subfraction MSA-7 (37.7 mg).

After each extraction and chromatographic separation step, the solvents were removed under reduced pressure using a rotary evaporator. The resulting extracts, fractions, and isolated compounds were subsequently kept under continuous airflow in a chemical fume hood to ensure complete evaporation of residual volatile solvents before storage and biological evaluation.

### 3.4 Analysis by UHPLC-HRMS/MS and Molecular Networking-Based Annotation

The crude extract (MSE) and the dichloromethane (MSD), ethyl acetate (MSA), and hydromethanolic (MSHM) fractions were analyzed using an ultrahigh-performance liquid chromatograph (Shimadzu Nexera X2) coupled to a high-resolution mass spectrometer equipped with a quadrupole time-of-flight (QTOF) analyzer (IMPACT II; Bruker Daltonics Corporation, USA) and an electrospray ionization source maintained at 40 °C.

The MSE, MSD, MSA, and MSHM samples were prepared in MeOH (1.0 mg mL^–1^), and chromatographic separation was performed using a C18 column (75 mm × 2.0 mm i.d.; 1.6 μm, Shim-pack XR-ODS III) with H_2_O (solvent A) and 0.1% formic acid in acetonitrile (solvent B) as the mobile phase. The gradient program was as follows: 5% B at 0–1 min, 30% B at 1–3 min, 95% B at 3–8 min, maintained at 95% B from 8 to 16 min, with a flow rate of 0.2 mL min^–1^ and an injection volume of 3 μL. The capillary voltage was set at 4500 V in positive and negative ionization modes, using sodium formate (10 μM) as a calibrator. The dry gas parameters were set at 8 L min^–1^ at 200 °C, with a nebulizer gas pressure of 4 bar. Collision-induced dissociation (CID) fragmentation was performed using argon (Ar) as the collision gas and a collision energy of 5–45 eV. Spectral data for the investigated samples were collected over an *m*/*z* range of 50–1300 and an acquisition rate of 5 spectra per second. Ions of interest were selected by MS/MS scan autofragmentation.

In GNPS2, the data were submitted for molecular networking using the classical_networking_workflow.^39^ The data were then clustered with MS-Cluster using a parent mass tolerance of 0.02 Da and an MS/MS fragment ion tolerance of 0.02 Da to create consensus spectra. A network was created using edge parameters of cosine scores above 0.7 and more than four matching peaks.

The data were filtered by removing all MS/MS peaks within ±17 Da of the *m*/*z* precursor. The network was then searched against the GNPS2 spectral libraries, and all matches were required to have a cosine score above 0.7 and at least four corresponding peaks. The network analysis was exported from GNPS2 and analyzed in Cytoscape,^40^ and the resulting data were also compared with the literature. Putative identification of the substances in the fractions was based on the fragmentation profiles of the generated spectra compared with literature data for previously identified compounds in the genus *Moquiniastrum* and with available databases.

The data obtained from the analyses were also processed using Data Analysis 4.3 software (Bruker). Accurate mass measurements were used to determine the molecular formulas of the compounds, considering an error of ≤10 ppm.^41^

### 3.5 Determination of the Relative Configuration Using pip-HSQMBC-IPAP NMR

The relative configuration of sordidic acid (**5**) was determined by analyzing the ^3^ *J*_CH_ coupling constants obtained from pip-HSQMBC-TOCSY-IPAP^42^ spectra. The analysis was carried out using a Bruker Avance III HD spectrometer operating at 500 MHz for ^1^H, with a 5.0 mm probe and the sample diluted in CDCl_3_. The ^3^ *J*_CH_ constants were selected for analysis because their behavior is analogous to that of ^3^ *J*_HH_ with respect to dihedral angles, as described by the Karplus curve. Typically, these constants range from 0 to 9 Hz, with the highest values observed when the C–C–C-H dihedral angle adopts an *anti*-configuration.

### 3.6 Antioxidant Assays

The antioxidant activities of the crude extract, fractions, and pure compounds were evaluated using two methods: ABTS (2,2′-azino-bis­(3-ethylbenzothiazoline-6-sulfonic acid)) and FRAP (ferric reducing antioxidant power). The free radical scavenging effect was evaluated using the ABTS assay.^43^ The ABTS^•+^ radical was prepared by mixing 7 mmol/L ABTS and 140 mmol/L potassium persulfate and then keeping the mixture in the dark for 16 h. The solution was subsequently diluted with ethanol to an absorbance of 0.70 ± 0.05. Aliquots of 30 μL (samples at 0.15–10 mg/mL) were reacted with 3.0 mL of the radical solution for 6 min in the dark. Absorbance was then recorded at 734 nm using a UV–visible spectrophotometer. A calibration curve was prepared using Trolox (0–2000 μmol/L). Results were expressed as μmol of Trolox equivalent (TE) per gram of sample (μmol TE/g). The FRAP assay^43,44^ was performed using aliquots (100 μL, in triplicate) of aqueous sample solutions (0.1–10 mg/mL), which were vortex-mixed with distilled water (300 μL) and FRAP reagent [3 mL; sodium acetate buffer solution (300 mmol/L, pH 3.6), 2,4,6-tripyridyl-*S*-triazine (TPTZ, 10 mmol/L), and FeCl_3_·6H_2_O (20 mmol/L), 10:1:1] and kept at 37 °C for 40 min before absorbance was measured at 593 nm using a UV–visible spectrophotometer. A calibration curve was prepared using FeSO_4_·7H_2_O (0–2000 μmol/L), and the iron-reducing power of the samples was expressed as μmol Fe­(II) equivalents per gram of sample (μmol Fe­(II)/g).

### 3.7 *In Vitro* Antiproliferative Assay

The antiproliferative activity against the HCT-116 human colon carcinoma cell line (ATCC Cat# CCL-247, RRID:CVCL_0291) was evaluated using the MTT cell viability assay. Crude extracts and fractions were initially diluted in DMSO (Synth, Brazil) to concentrations of 10 and 1 mg mL^–1^. Subsequently, 1 × 10^4^ cells per well were seeded in 96-well plates (corresponding to 5 × 10^4^ cells mL^–1^ in 200 μL of medium). After 24 h of incubation, the test samples were added at final concentrations of 5 and 50 μg mL^–1^ (in duplicate) and incubated for an additional 72 h. Following this period, the supernatant was replaced with fresh culture medium containing MTT (0.5 mg mL^–1^) (Sigma-Aldrich, USA). After a 3-h incubation, the supernatant was discarded, the plates were dried, and the resulting blue formazan crystals were dissolved in 150 μL of DMSO. Absorbance was then measured at 570 nm.^45^ The percentage of cell growth inhibition for each sample was calculated using GraphPad Prism v10.0 software. Doxorubicin (Sigma-Aldrich, USA) was used as the positive control and DMSO as the negative control. Final data represent the mean ± standard deviation of three independent experiments (*n* = 3).

### 3.8 Antimicrobial Activity

The antimicrobial assay was performed using the microdilution method to determine the minimum inhibitory concentration (MIC) in sterile 96-well microplates, according to the Clinical and Laboratory Standards Institute.^46^ The antimicrobial activities of MSE and the MSH, MSD, MSA, and MSHM fractions were tested against Gram-positive [*S. aureus* (ATCC 25923), *B. cereus* (ATCC 11778)] and Gram-negative [*S. enterica* subsp. *enterica* serovar Enteritidis (ATCC 13076), *E. coli* (ATCC 25922)] bacterial strains.

Aliquots of the crude extract (MSE) and the hexane (MSH), dichloromethane (MSD), ethyl acetate (MSA), and hydromethanolic (MSHM) fractions were initially diluted to a concentration of 10 mg/mL, while pure compounds were diluted to 1 mg/mL, followed by serial dilution (1:2) in Mueller-Hinton (MH) medium in 96-well plates (100 μL/well, final volume). Each bacterial inoculum was adjusted to 0.5 on the McFarland scale (turbidity = 0.5, approximately 1.5 × 10^8^ CFU/mL) in sterile 0.85% NaCl solution, followed by dilution in MH medium (1:20, v/v). After sample dilution in the 96-well plates, each bacterial suspension (10 μL/well, one bacterial strain per plate) was inoculated and incubated at 35 °C ± 2 °C for 24 h. Ampicillin was used as a positive control at a concentration of 1 mg/mL. The lowest concentration of each sample required to inhibit microbial growth (MIC) was then determined by visual observation and confirmed using 0.5% 2,3,5-triphenyltetrazolium chloride (TTC) solution. The minimum bactericidal concentration (MBC) was determined as the lowest concentration yielding negative subcultures on MH agar from wells showing no growth. These tests were performed in triplicate.

### 3.9 Antileishmanial Activity

The promastigote forms of *L. amazonensis* used in this work correspond to the IFLA/BR/1967/PH8 strain transfected with the pIR1SAT-gene LUC­(a)­DsRed2­(b). Parasites were kept at 25 °C in Warren medium (brain heart infusion, hemin, and folic acid, pH 7.2) containing 10% heat-inactivated fetal bovine serum (FBS). The J774A.1 macrophage lineage (ATCC TIB-67) was grown in RPMI 1640 medium (pH 7.6) supplemented with l-glutamine and 10% FBS, under a 5% CO_2_ atmosphere at 37 °C.

For the promastigote-stage assay, parasites harvested after 72 h of growth were added to 96-well plates in the presence and absence of the compounds, diluted in 1% DMSO, and then incubated for 72 h at 25 °C. Treatments were carried out at final concentrations of 1–200 μg mL^–1^, and parasite viability was assessed by the XTT reduction assay.^47^ After the treatment period, 0.5 mg mL^–1^ XTT solution was added and, following a 4 h incubation, the absorbance at 450 nm was recorded on a Power Wave XS microplate reader (BioTek). The concentrations that inhibited 50% of the parasites (IC_50_) were determined by nonlinear regression of the plotted values.

Intracellular amastigotes were obtained by coculturing promastigotes (5 × 10^6^ mL^–1^) with macrophages (5 × 10^5^ mL^–1^; 1:10 parasite-to-cell ratio) in white 96-well plates. After 24 h at 34 °C and 5% CO_2_, a period sufficient for parasite internalization and differentiation into amastigotes, the cells were washed to eliminate noninternalized parasites and treated with increasing concentrations of the substances (1–100 μg mL^–1^) for 48 h. Amastigote viability was then quantified with the Pierce Firefly Luc One-Step Glow Assay kit (Thermo Fisher, Rockford, USA), following the supplier’s protocol. Luminescence was quantified using a SpectraMaxL luminometer (Molecular Devices) with λ_em_ = 570 nm and an integration time of 1 s. Inhibition percentages were calculated based on the luminescence of the untreated control, and IC_50_ values were calculated by nonlinear regression.^48^ All results are reported as the mean ± standard deviation from at least three independent experiments.

The cytotoxic potential of the compounds toward host cells was determined with the MTT reduction assay using J774A.1 macrophages.^45^ Cells (5.0 × 10^5^ mL^–1^), maintained in RPMI medium with 10% FBS, were distributed into 96-well plates and allowed to adhere for 24 h at 37 °C under 5% CO_2_. The compounds were then applied at concentrations from 10 to 1000 μg mL^–1^ for 48 h. After treatment, the cells were washed with PBS (pH 7.2) and incubated in the presence of MTT (2 mg mL^–1^) for 4 h. The supernatant was removed, the formazan crystals were solubilized in DMSO, and absorbance was measured at 570 nm using a plate spectrophotometer (Power Wave XS; BioTek). Cell viability was calculated relative to the control. CC_50_ values (50% cytotoxic concentration) were determined by nonlinear regression analysis. Data correspond to the mean ± standard deviation of at least three independent experiments.

## Supplementary Material



## Notes

Structure and ^1^H and ^13^C NMR data of simiarenol (1); ^1^H NMR spectrum with expansion (CDCl_3_, 500 MHz) of compound 5; molecular network was constructed from 317 precursor ions detected in the negative ionization mode from UPLC-MS/MS data of *M. sordidum*; inhibition rate (%) of samples submitted for testing against colon carcinoma cell line (HCT-116) at of 5 and 50 μg mL^–1^ concentrations (PDF)
