# Supplementary material for: Two New Metabolites from Moquiniastrum sordidum: Phytochemical Characterization and Multifaceted Bioactivity
Source: ACS Omega. 2026 Oct 20;11(43):66618–32. doi: 10.1021/acsomega.6c06069 (PMC13737026; doi:10.1021/acsomega.6c06069)
Supplement: Supplementary file 1 [file ao6c06069_si_001.pdf]

Two New Metabolites from *Moquiniastrum sordidum*: Phytochemical Characterization and Multifaceted Bioactivity

*Tamires Cordeiro[a], Francielli A. P. Valeze[a], Rodolfo B. Balbinot[b], Bianca D. B. Sahm[c], Fernanda F. Dourado[a], Elizabeth F. Pires[d], Aline Pivetta[d], Tatiana S. Tiuman[d], Celso V. Nakamura[b], Leticia V. Costa-Lotufo[c], Marta R. B. do Carmo[e], Renan V. Viesser[a], Ernani A. Basso[a], Maria H. Sarragiotto[a] and Debora C. Baldoqui[a, *].*

[a]Departamento de Química, Universidade Estadual de Maringá, Av. Colombo 5790, Maringá, PR, Brazil; [b] Programa de Pós-Graduação em Ciências Biológicas, Universidade Estadual de Maringá, Av. Colombo 5790, 87020-900, Maringá, Paraná, Brazil; [c]Instituto de Ciências Biomédicas, Universidade de São Paulo, Av. Prof. Lineu Prestes, 1524, São Paulo, SP, Brazil; [d]Universidade Tecnológica Federal do Paraná, R. Cristo Rei, 19, 85902-490, Toledo, PR, Brazil. [e]Departamento de Biologia Geral, Universidade Estadual de Ponta Grossa, Av. Carlos Cavalcanti, 4748, Ponta Grossa, PR, Brazil

Supporting information

Table of contents

Figure S1 - Structure and ^{1}H and ^{13}C NMR data of simiarenol (**1**).

Figure S2 - Structure and ^{1}H and ^{13}C NMR data of oleanolic acid (**2**).

Figure S3 - Structure and ^{1}H and ^{13}C NMR data of hispidulin (**3**).

Figure S4 - Structure and ^{1}H and ^{13}C NMR data of tyrosol (**4**).

Figure S5 - Structure and ^{1}H and ^{13}C NMR data of genkwanin (**6**).

Figure S6 - Structure and ^{1}H and ^{13}C NMR data of apigenin (**7**).

Figure S7 - ^{1}H-NMR spectrum with expansion ($CDCl_3$, 500 MHz) of compound **5**.

Figure S8 - ^{13}C-NMR spectrum with expansion ($CDCl_3$, 125 MHz) of compound **5**.

Figure S9 - ^{1}H-^{13}C HMBC spectrum with expansion ($CDCl_3$, 500 and 125 MHz) for **5**.

Figure S10 - ^{1}H-^{13}C HSQC spectrum ($CDCl_3$, 500 and 125 MHz) for **5**.

Figure S11 - COSY spectrum ($CDCl_3$, 500 and 125 MHz) for **5**.

Figure S12 - ^{1}H-NMR spectrum with expansion (DMSO-d_6,300 MHz) of compound **8**.

Figure S13 - ^{13}C-NMR spectrum with expansion (DMSO-d_6, 75 MHz) of compound **8**.

Figure S14 - ^{1}H-^{13}C HSQC spectrum (DMSO-d_6, 300 and 75 MHz) for **8**.

Figure S15 - ^{1}H-^{13}C HMBC spectrum with expansion (DMSO-d_6, 300 and 75 MHz) for **8**.

Figure S16 - COSY spectrum (DMSO-d_6. 300 MHz) of compound **8**.

Figure S17 - Molecular network was constructed from 317 precursor ions detected in the negative ionization mode from UPLC-MS/MS data of *M. sordidum.*

Table S1. Inhibition rate (%) of samples submitted for testing against colon carcinoma cell line (HCT-116) at of 5 and 50 μg mL^{-1} concentrations.

Structures and spectroscopic data of compounds **1-4** and **6-7** isolated from aerial parts of *M. sordidum.*

Figure S1 - Structure and ^{1}H and ^{13}C NMR data of simiarenol (**1**).

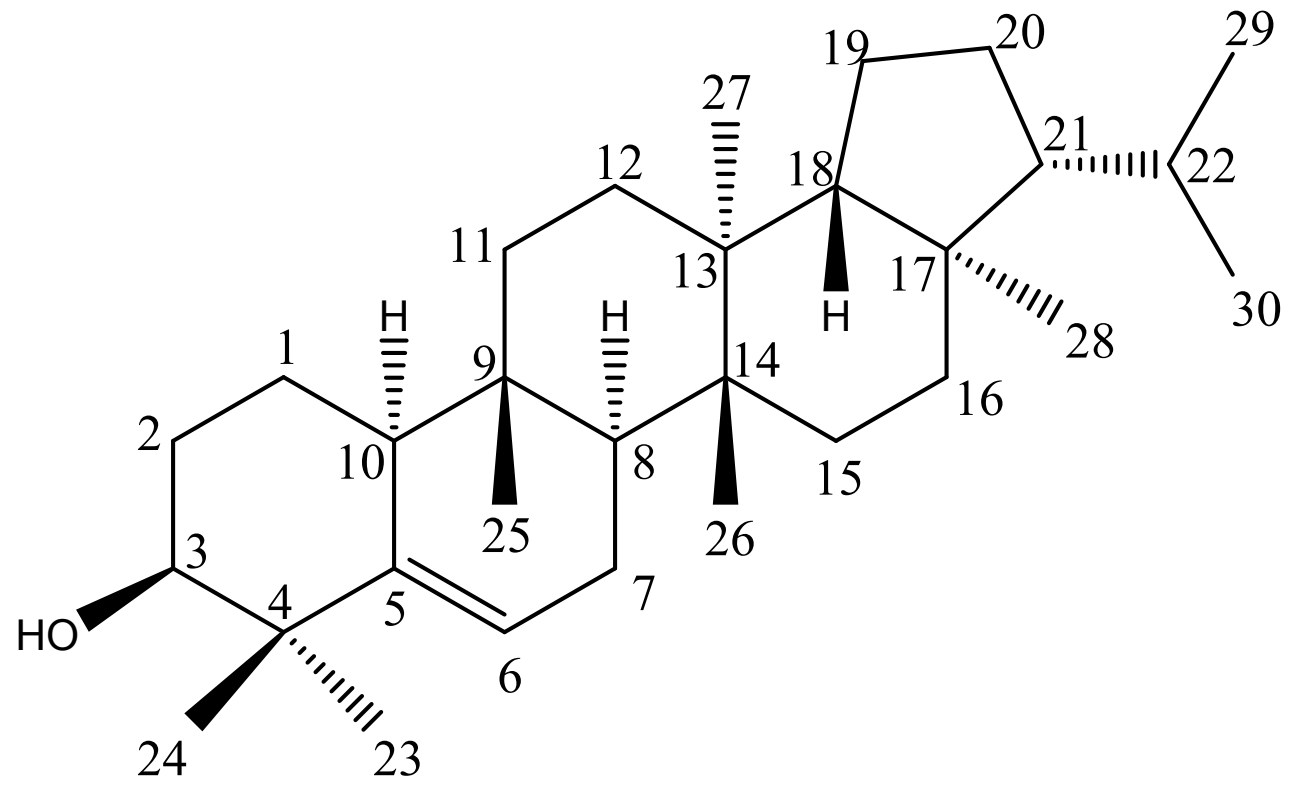

^{1}H NMR (300 MHz, DMSO-d$_6$): δ 5.62 (1H, *m*, H-6), 3.47 (1H, *m*, *J* = 2.7 and 5.6 Hz, H-3), 1.14 (3H, *s*, H-24), 1.05 (3H, *s*, H-23), 1.00 (3H, *s*, H-26), 0.93 (3H, *s*, H-27), 0.90 (3H, *s*, H-25), 0.88 (3H, *s*, H-29), 0.84 (3H, *m*, H-30), 0.78 (3H, *s*, H-28). ^{13}C NMR (75 MHz, DMSO-d$_6$): δ 142.0 (C-5), 122.0 (C-6), 76.4 (C-3), 60.1 (C-21), 51.8 (C-18), 50.3 (C-10), 44.3 (C-8), 42.8 (C-17), 40.8 (C-4), 39.5 (C-14), 38.6 (C-13), 35.4 (C-16), 34.8 (C-9), 34.2 (C-11), 30.8 (C-22), 29.1 (C-15), 29.1 (C-23), 29.0 (C-12), 28.3 (C-20), 27.8 (C-2), 25.5 (C-24), 24.1 (C-7), 22.9 (C-30), 21.9 (C-29), 19.9 (C-19), 18.1 (C-1), 17.9 (C-25), 16.1 (C-28), 15.8 (C-26), 15.0 (C-27).

Figure S2 - Structure and ^{1}H and ^{13}C NMR data of oleanolic acid (**2**).

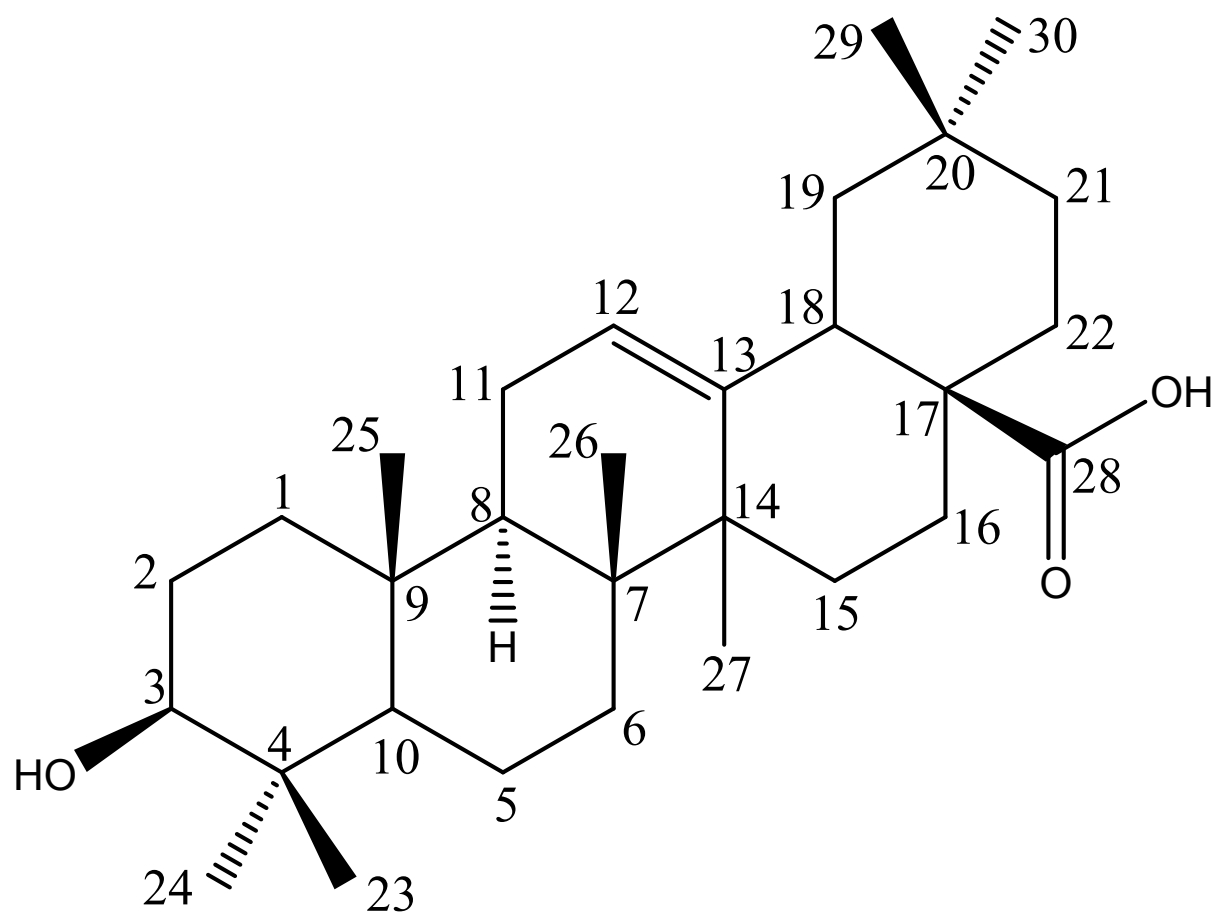

^{1}H NMR (300 MHz, DMSO-d$_6$): δ 5.28 (1H, *t*, *J* = 3.6 Hz, H-12), 3.22 (1H, *dd*, *J* = 4.9 and 10.6 Hz, H-3), 1.14 (3H, *s*, H-27), 0.91 (1H, *d*, H-8), 0.76 (2H, *d*, H-5). ^{13}C NMR (75 MHz, DMSO-d$_6$): δ 183.2 (C-28), 143.8 (C-13), 122.8 (C-12), 79.2 (C-3), 55.4 (C-5), 47.8 (C-9), 46.7 (C-17), 46.1 (C-19), 41.8 (C-14), 41.2 (C-18), 39.5 (C-8), 38.9 (C-4), 38.5 (C-1), 37.2 (C-10), 34.0 (C-21), 32.6 (C-7), 32.6 (C-22), 30.9 (C-20), 28.3 (C-23), 27.9 (C-15), 27.4 (C-2), 26.1 (C-27), 23.8 (C-30), 23.6 (C-16), 23.1 (C-11), 18.5 (C-6), 17.2 (C-26), 15.7 (C-24), 15.5 (C-25).

Figure S3 - Structure and ^{1}H and ^{13}C NMR data of hispidulin (**3**).

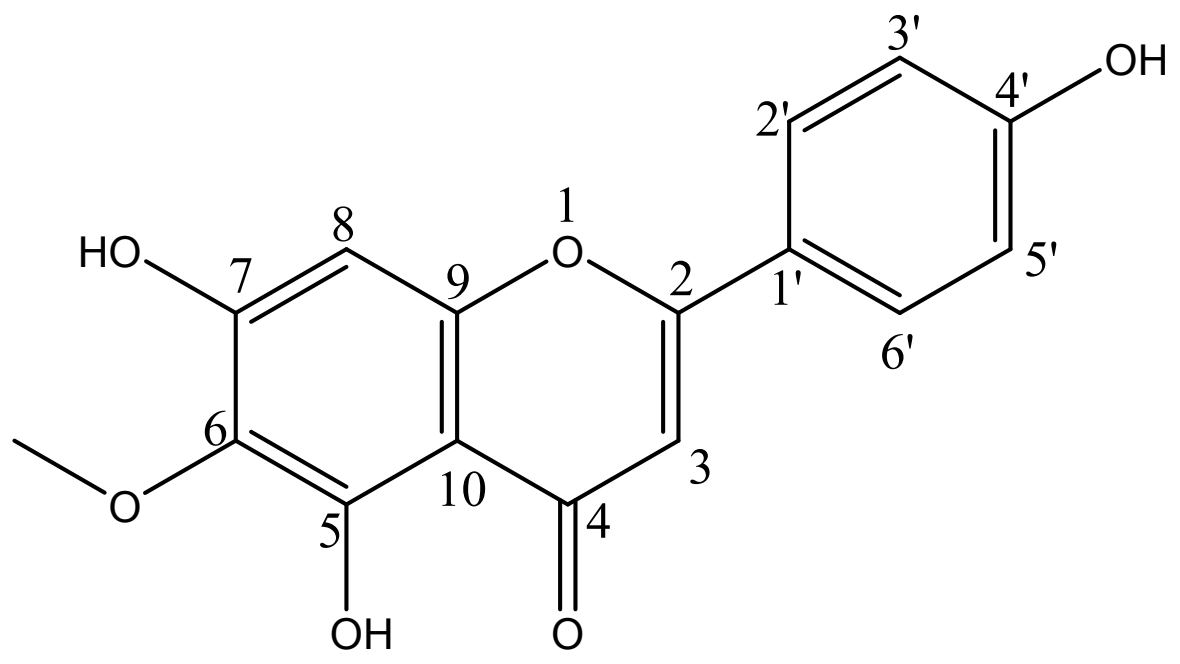

^{1}H NMR (300 MHz, DMSO-d_6): δ 7.87 (2H, *d*, *J* = 8.8 Hz, H-2'/6'), 6.91 (2H, *d*, *J* = 8.8 Hz, H-3'/5'), 6.76 (1H, *s*, H-3), 6.58 (1H, *s*, H-8), 3.73 (3H, *s*, OCH_3). ^{13}C NMR (75 MHz, DMSO-d_6): δ 182.8 (C-4), 164.5 (C-2), 161.8 (C-4'), 157.9 (C-7), 153.4 (C-5), 153.0 (C-9), 131.9 (C-6), 128.9 (C-2'/6'), 121.8 (C-1'), 115.6 (C-3'/5'), 104.7 (C-10), 103.0 (C-3), 94.9 (C-8), 60.6 (OCH_3).

Figure S4 - Structure and ^{1}H and ^{13}C NMR data of tyrosol (**4**).

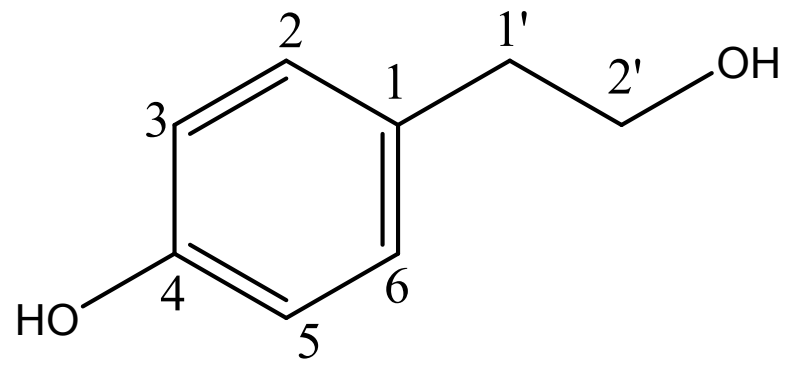

^{1}H NMR (300 MHz, DMSO-d_6): δ 9.11 (1H, *brs*, 4-OH), 6.96 (2H, *d*, *J* = 8.6 Hz, H-2/6), 6.63 (2H, *d*, *J* = 8.6 Hz, H-3/5), 4.56 (1H, *brs*, 1'-OH), 3.51 (2H, *t*, *J* = 7.2 Hz, H-1'), 2.58 (2H, *t*, *J* = 7.2 Hz, H-2'). ^{13}C NMR (75 MHz, DMSO-d_6): δ 156.1 (C-4), 129.9 (C-2/6), 129.1 (C-1), 115.6 (C-3/5), 63.2 (C-1'), 38.9 (C-2').

Figure S5 - Structure and ^{1}H and ^{13}C NMR data of genkwanin (**6**).

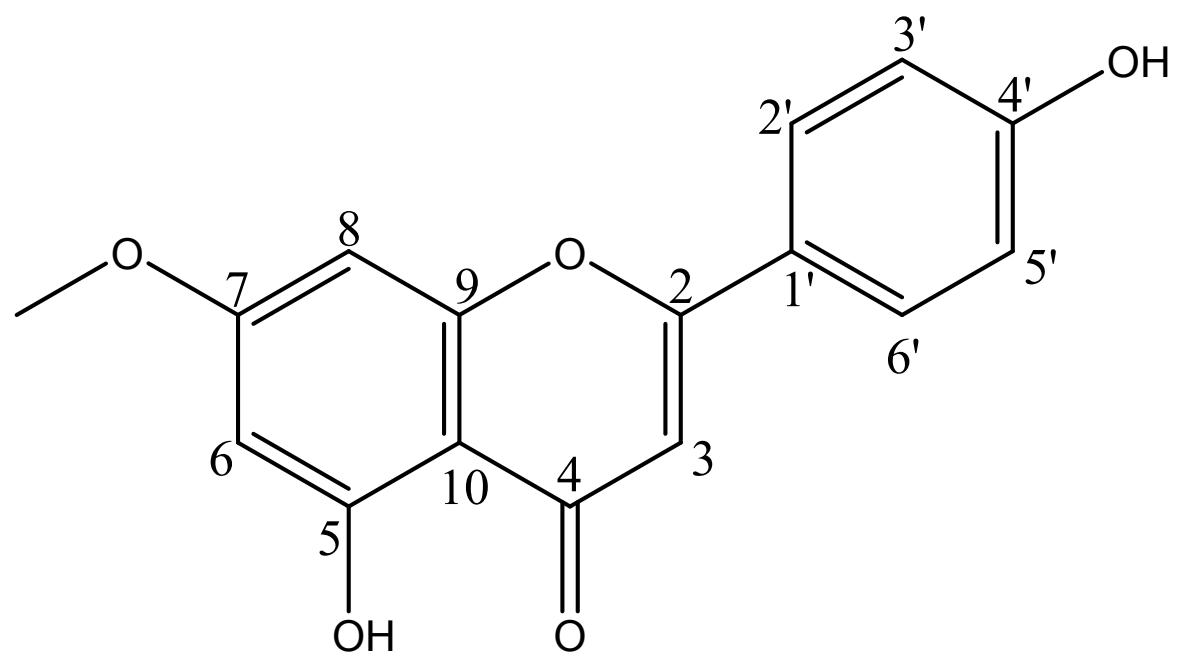

^{1}H NMR (300 MHz, DMSO-d_6): δ 7.93 (2H, *d*, *J* = 8.8 Hz, H-2'/6'), 6.91 (2H, *d*, *J* = 8.8 Hz, H-3'/5'), 6.81 (1H, *s*, H-3), 6.74 (1H, *d*, H-8), 6.35 (1H, *d*, H-6), 3.84 (3H, *s*, OCH_3). ^{13}C NMR (75 MHz, DMSO-d_6): δ 182.6 (C-4), 165.8 (C-7), 164.9 (C-2), 161.9 (C-4'), 161.8 (C-5), 157.9 (C-9), 129.2 (C-2'/6'), 121.6 (C-1'), 116.8 (C-3'/5'), 105.3 (C-10), 103.7 (C-3), 98.6 (C-6), 93.8 (C-8), 57.0 (OCH_3).

Figure S6 - Structure and ^{1}H and ^{13}C NMR data of apigenin (**7**).

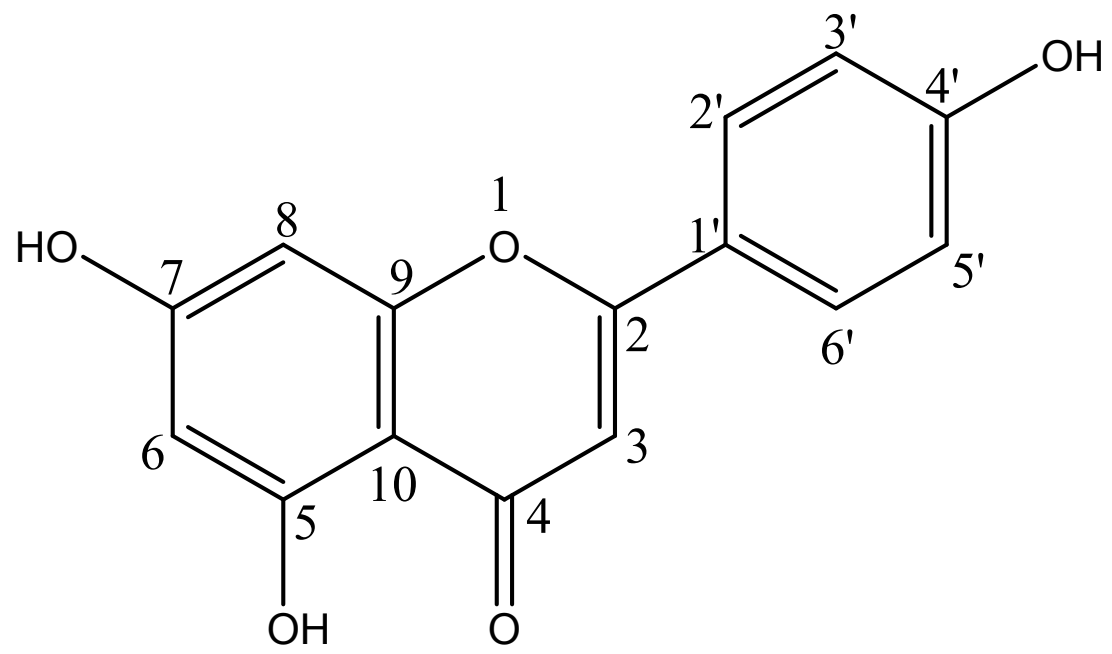

^{1}H NMR (300 MHz, DMSO-d_6): δ 12.90 (1H, *s*, 5-OH), 7.91 (2H, *d*, *J* = 8.9 Hz, H-2'/6'), 6.91 (2H, *d*, *J* = 8.9 Hz, H-3'/5'), 6.76 (1H, *s*, H-3), 6.47 (1H, *d*, *J* = 2.1 Hz, H-8), 6.18 (1H, *d*, *J* = 2.1 Hz, H-6).

Figure S7 - ^{1}H-NMR spectrum with expansion ($CDCl_3$, 500 MHz) of compound **5**.

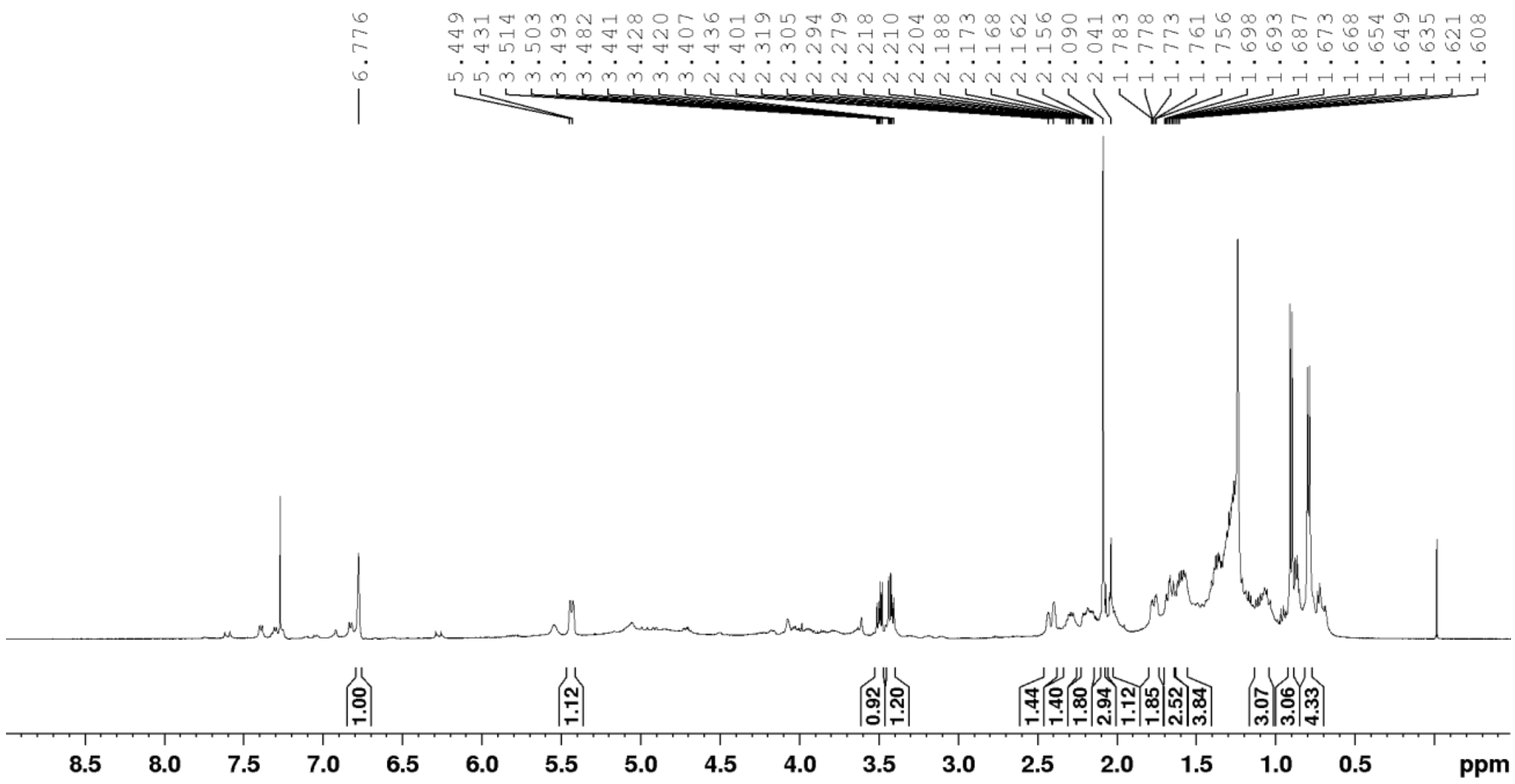

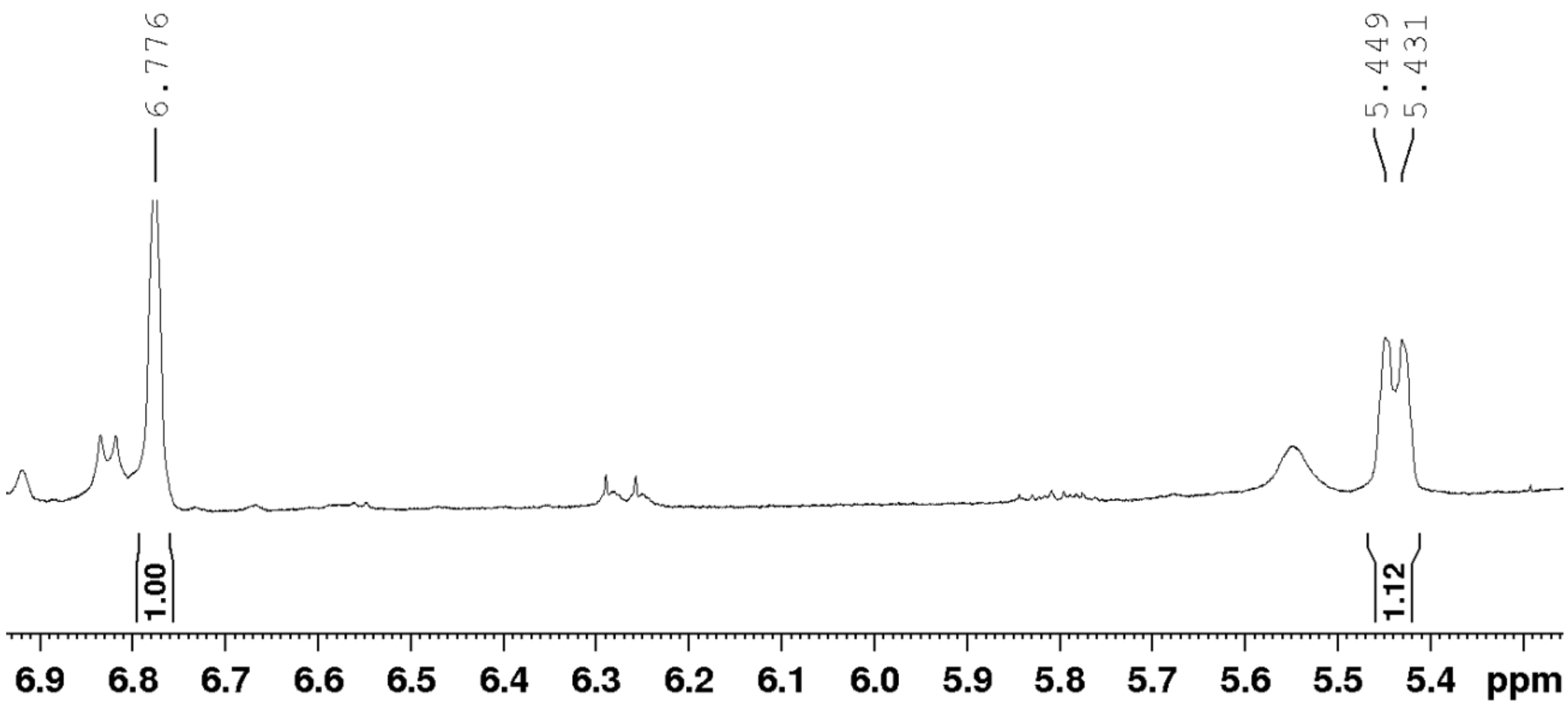

Figure S7 - ^{1}H-NMR spectrum with expansion ($CDCl_3$, 500 MHz) of compound **5** - continuation

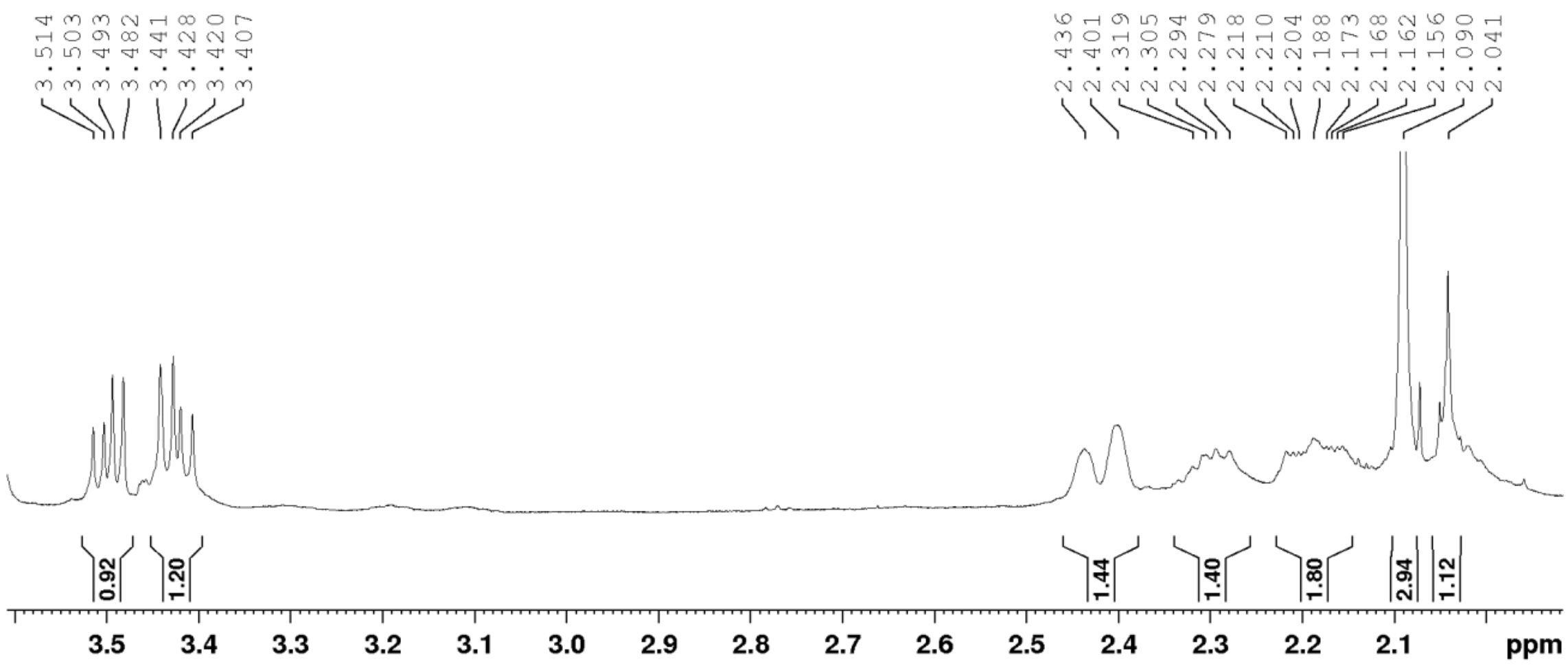

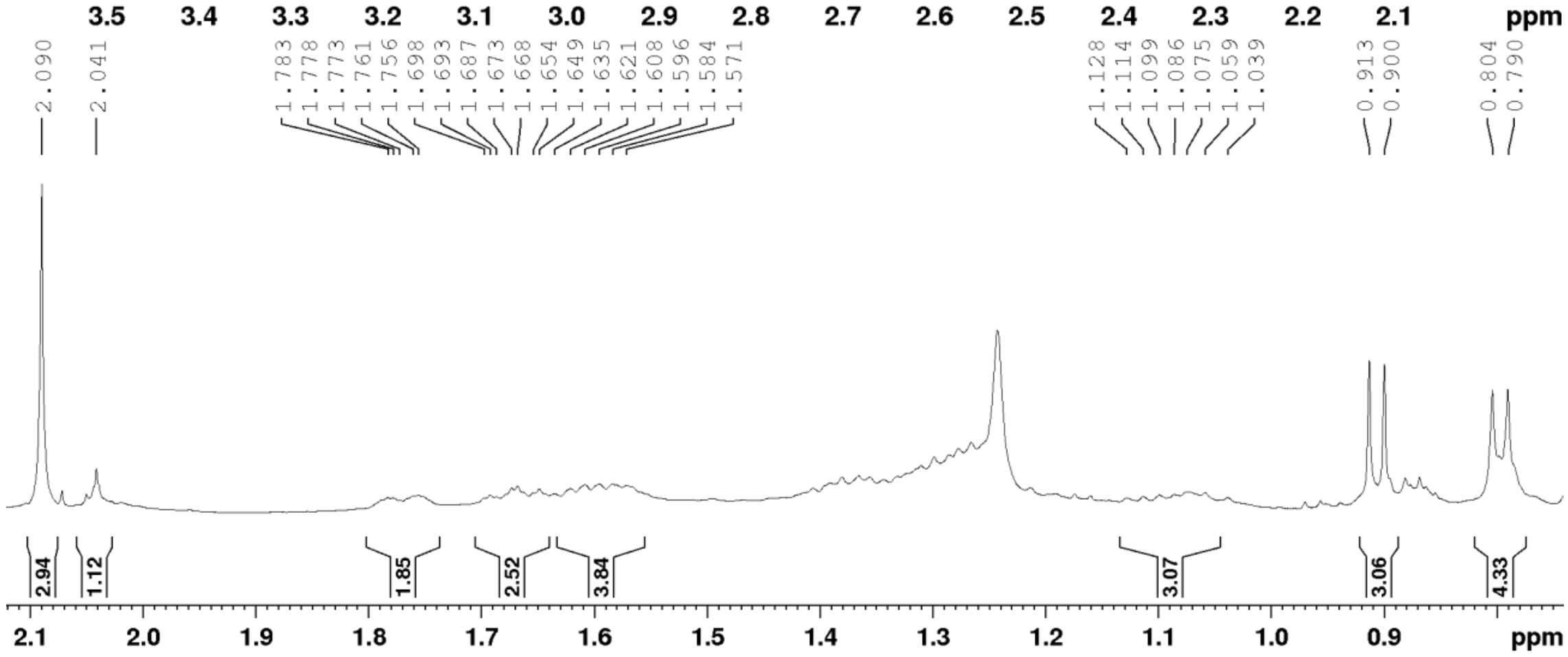

Note: The integrations in the aliphatic region include contributions from minor impurities and overlapping signals.

Figure S8 - ^{13}C-NMR spectrum with expansion ($CDCl_3$, 125 MHz) of compound **5**.

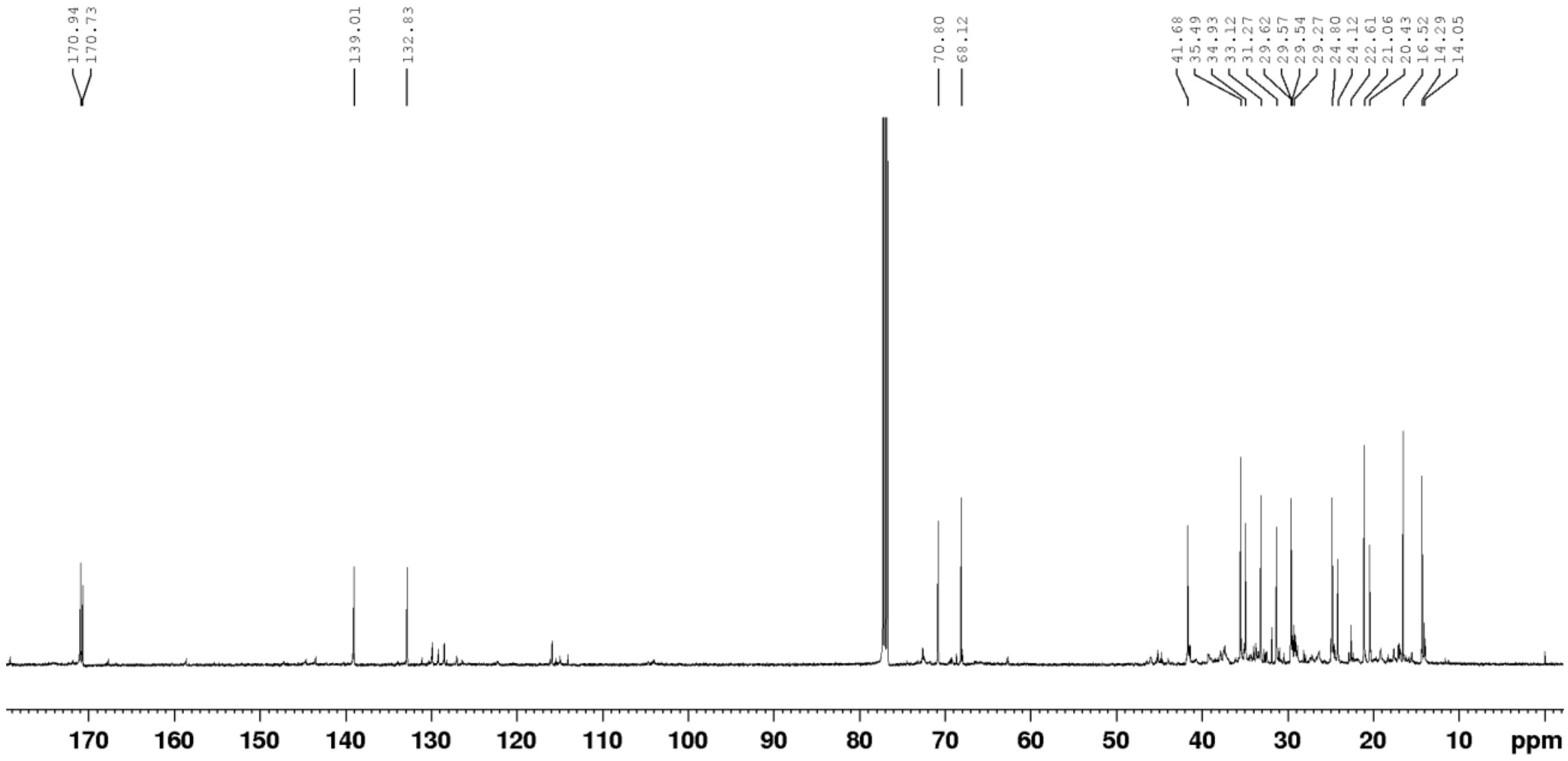

Figure S8 - ^{13}C-NMR spectrum with expansion ($CDCl_3$, 125 MHz) of compound **5** – continuation.

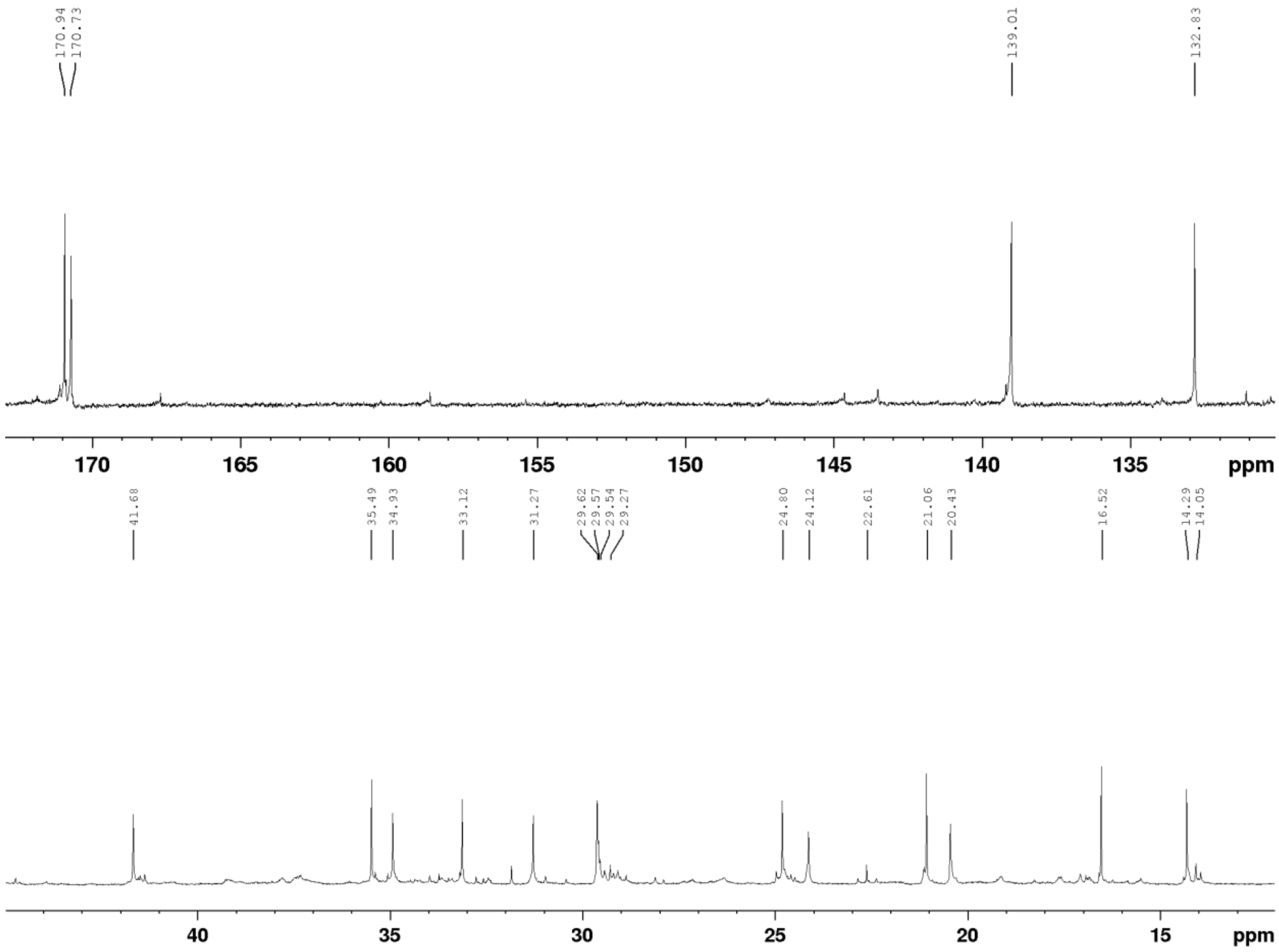

Figure S9 - ^{1}H-^{13}C HMBC spectrum with expansion ($CDCl_3$, 500 and 125 MHz) for **5**.

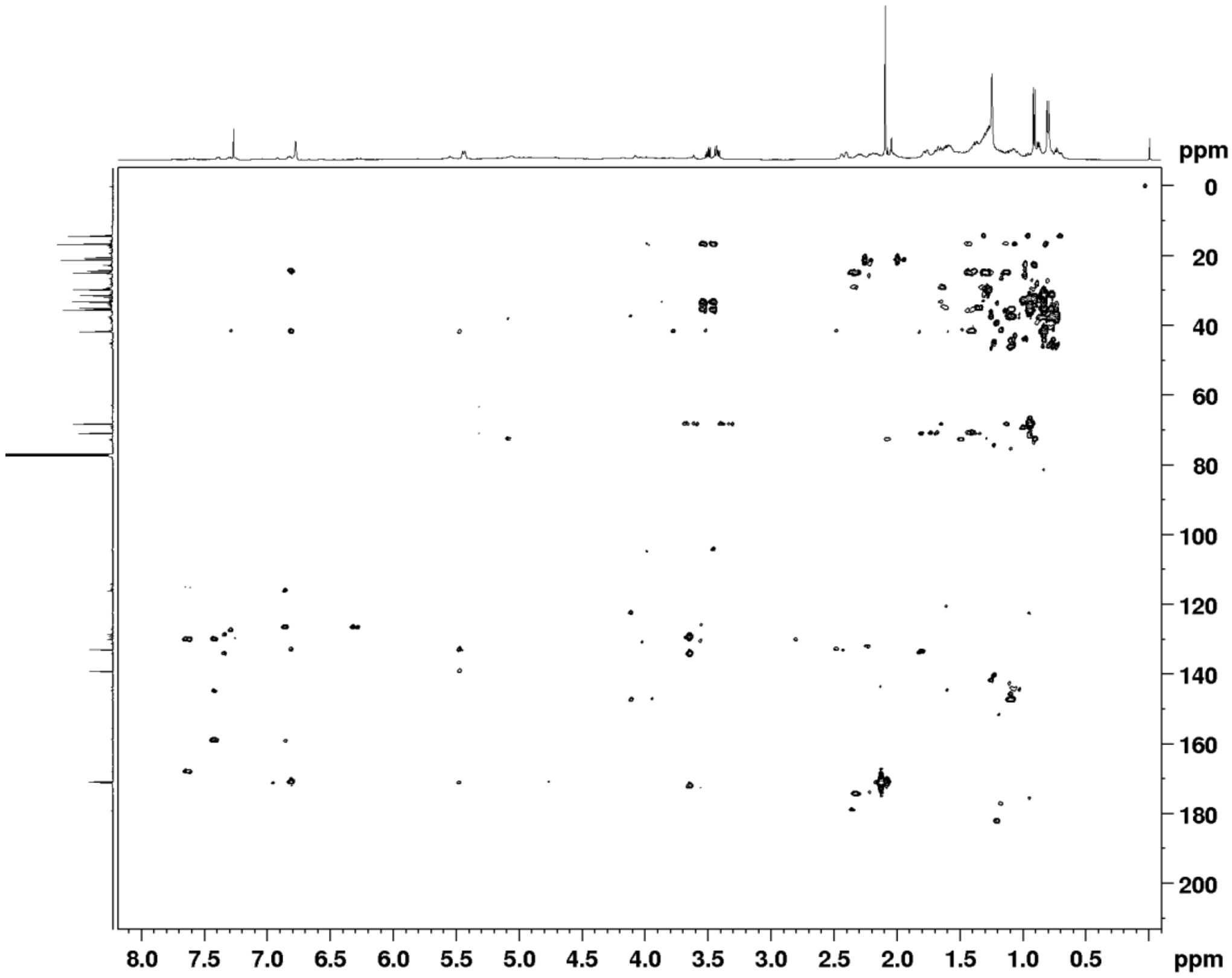

Figure S9 - ^{1}H-^{13}C HMBC spectrum with expansion ($CDCl_3$, 500 and 125 MHz) for **5** – continuation.

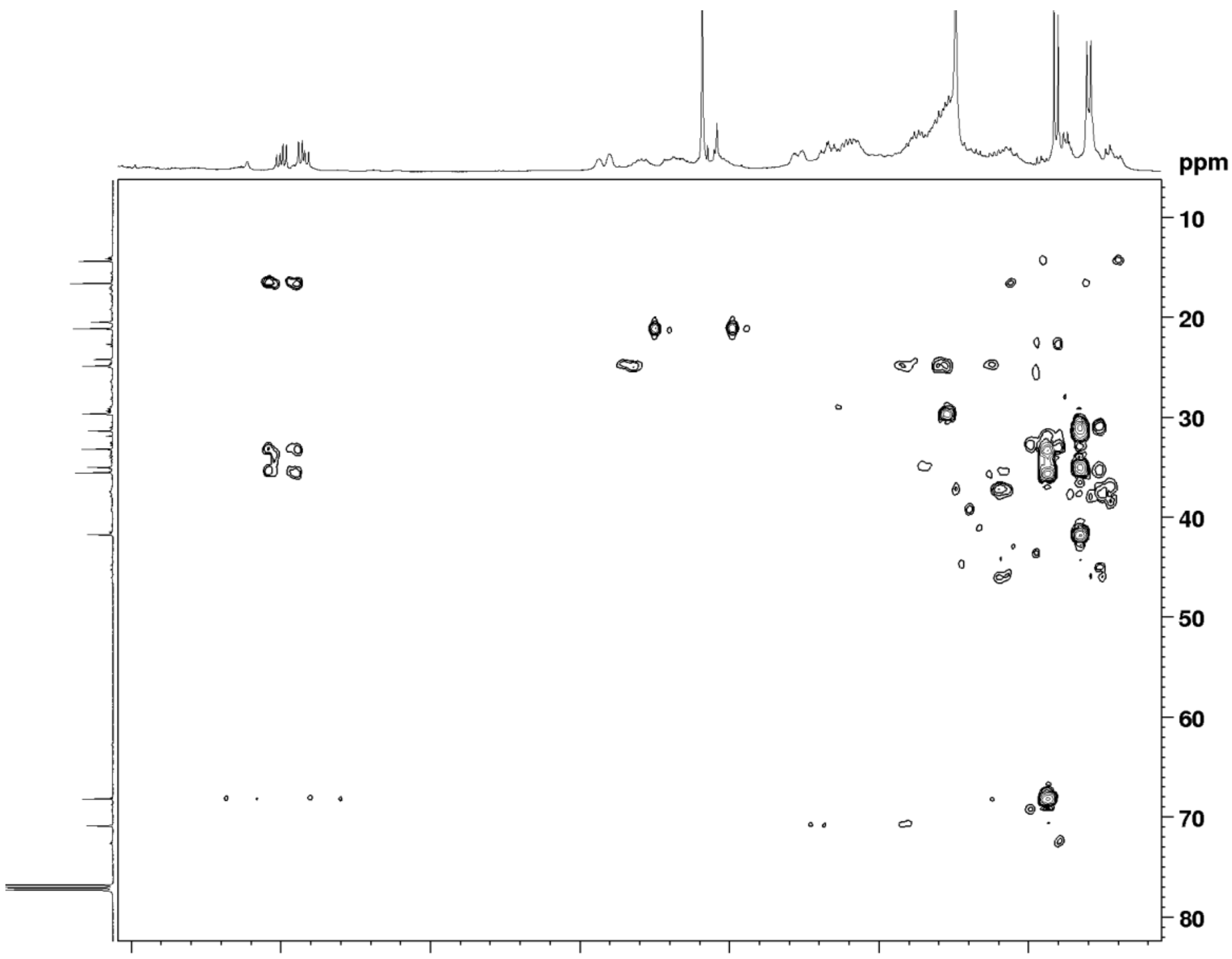

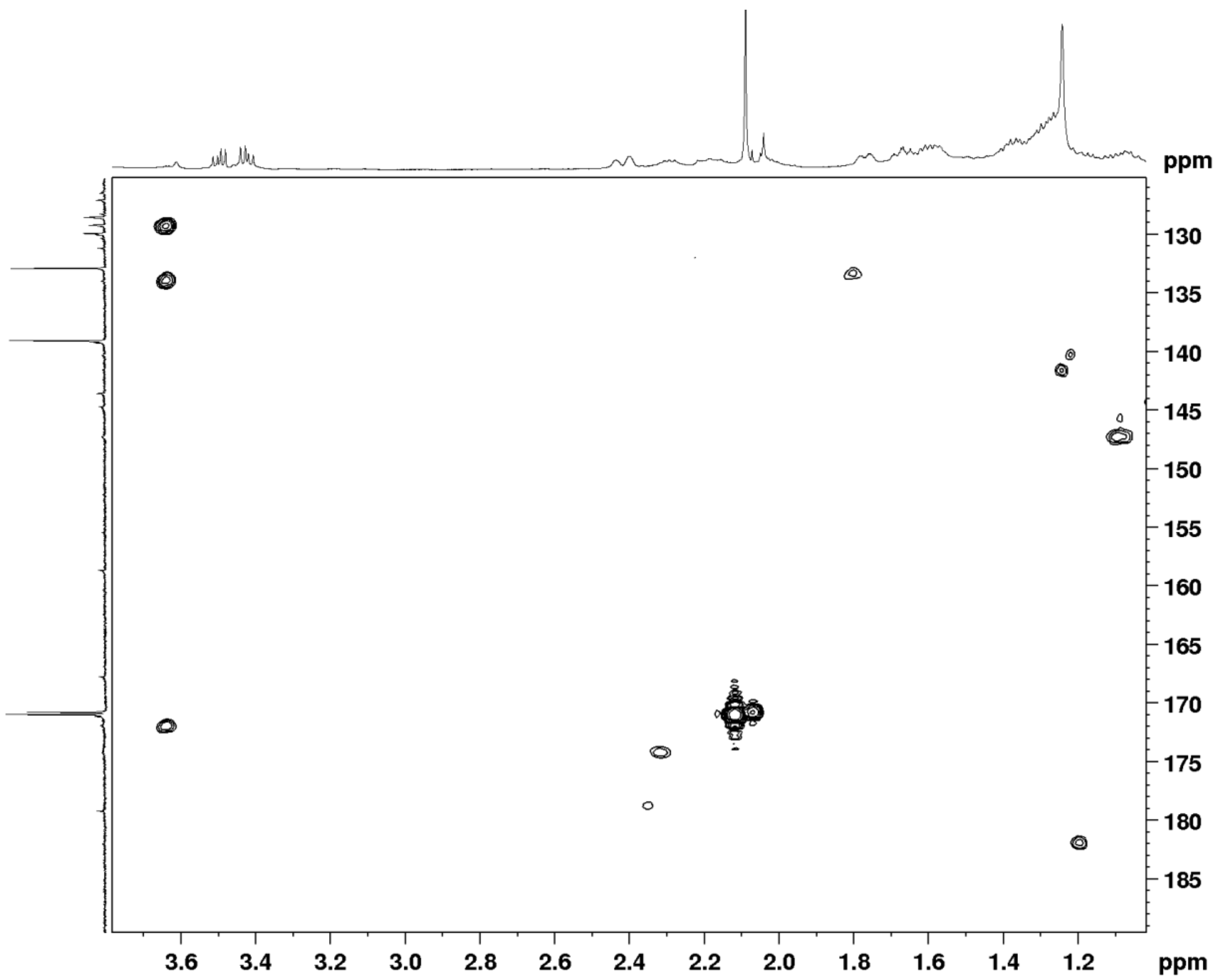

Figure S9 - ^{1}H-^{13}C HMBC spectrum with expansion ($CDCl_3$, 500 and 125 MHz) for **5** – continuation.

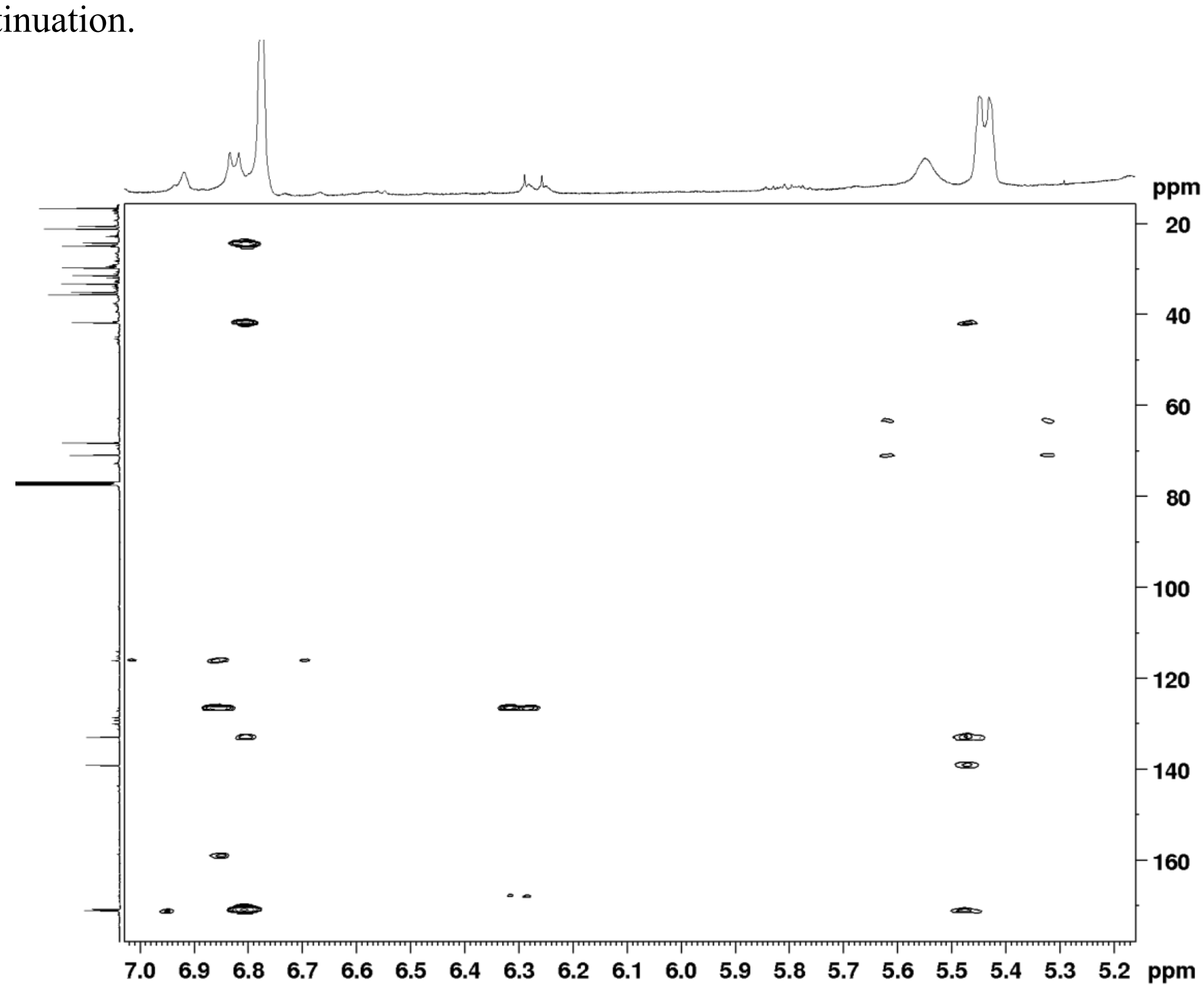

Figure S10 - ^{1}H-^{13}C HSQC spectrum ($CDCl_3$, 500 and 125 MHz) for **5**.

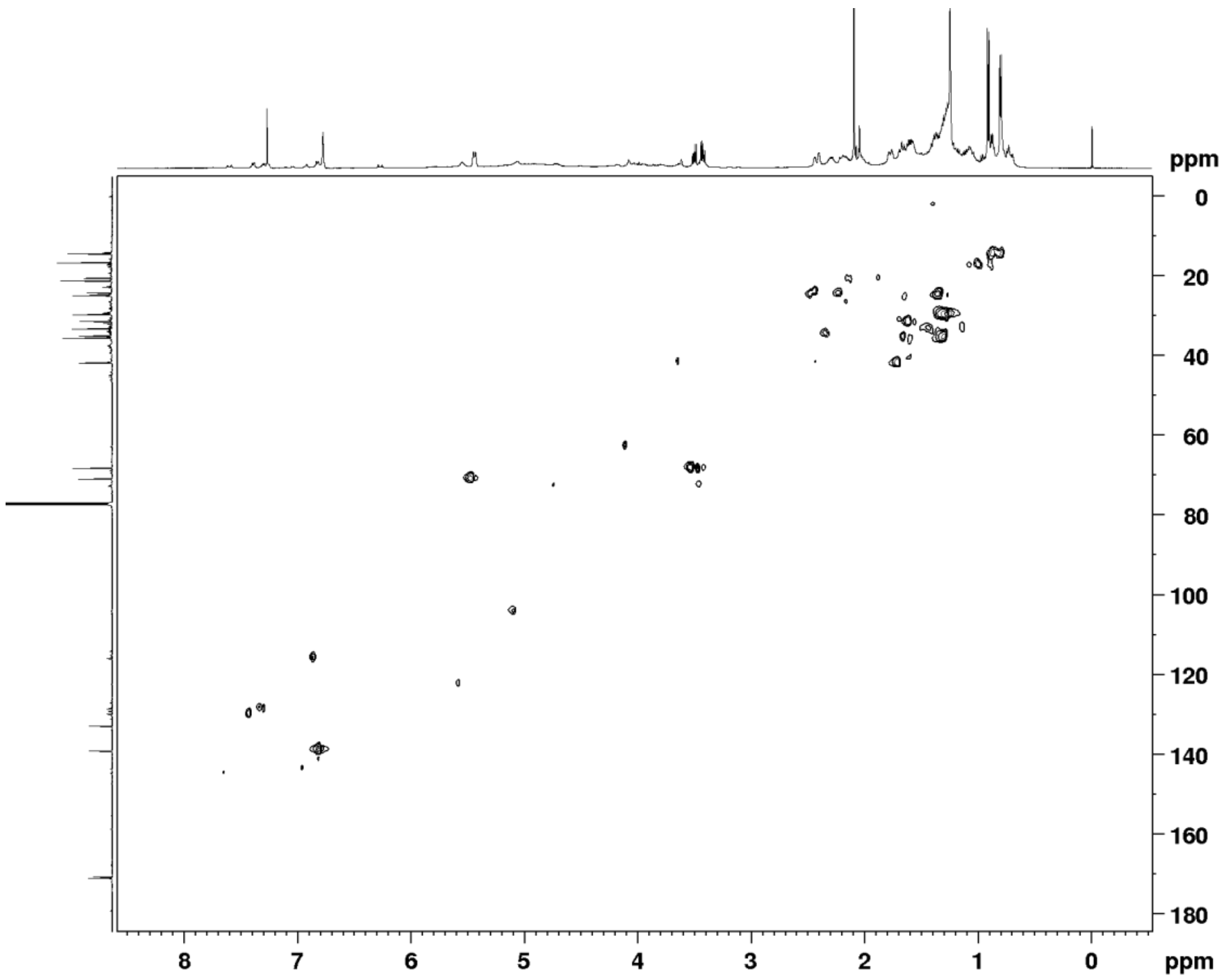

Figure S11 - COSY spectrum ($CDCl_3$, 500 and 125 MHz) for **5**.

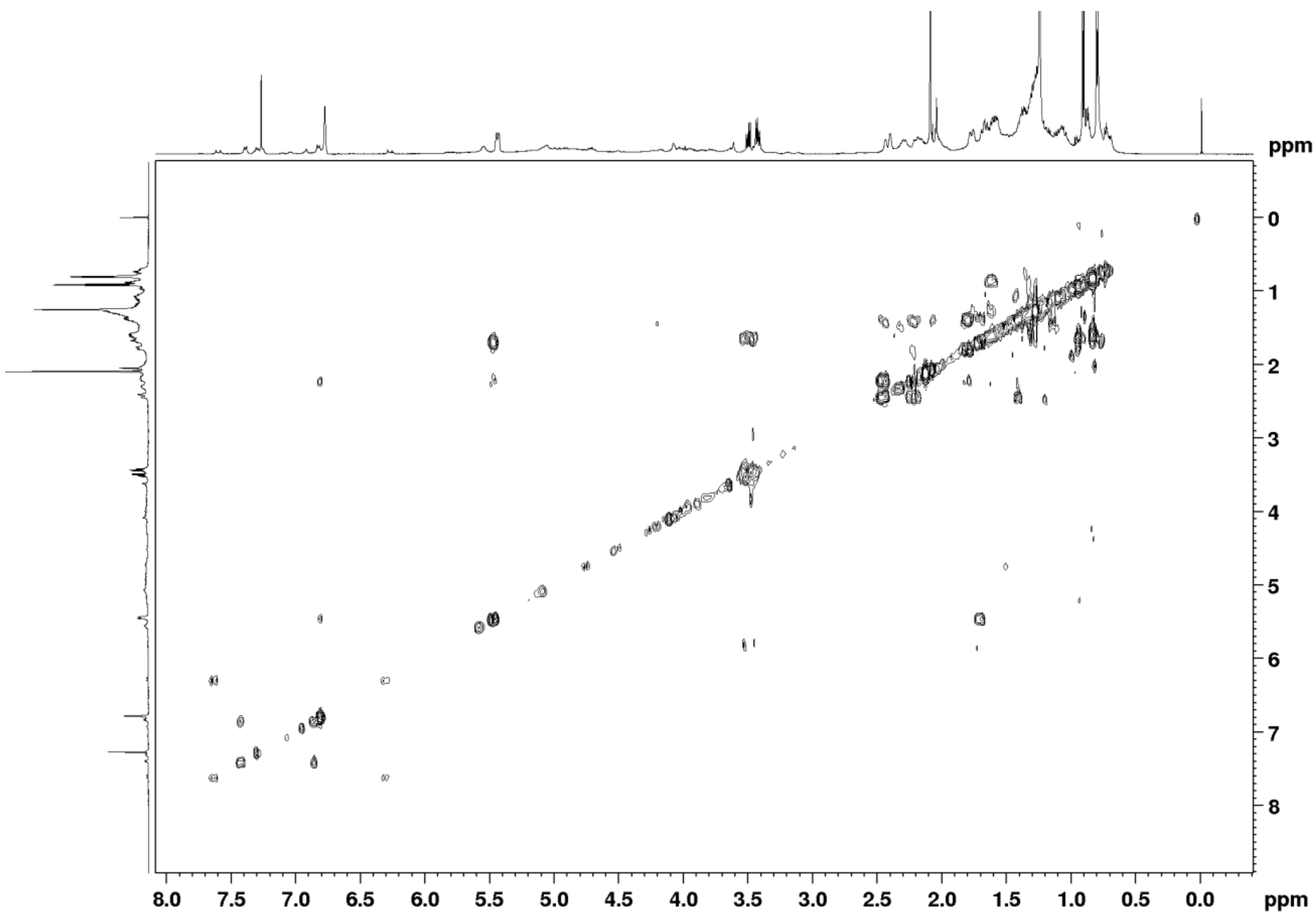

Figure S12 - ^{1}H-NMR spectrum with expansion (DMSO-d_6,300 MHz) of compound **8**.

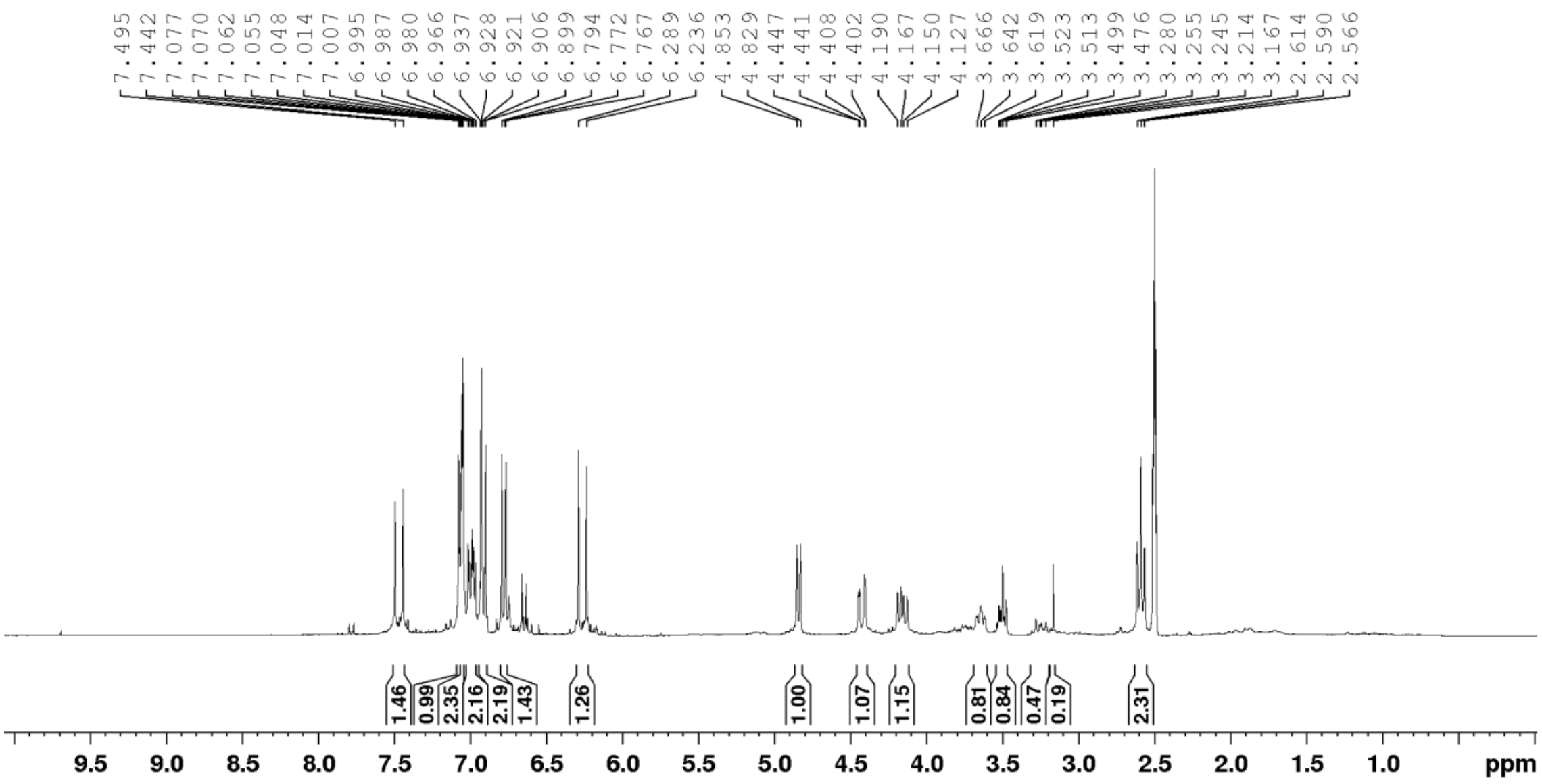

Figure S12 - ^{1}H-NMR spectrum with expansion (DMSO-d_6,300 MHz) of compound **8** - continuation.

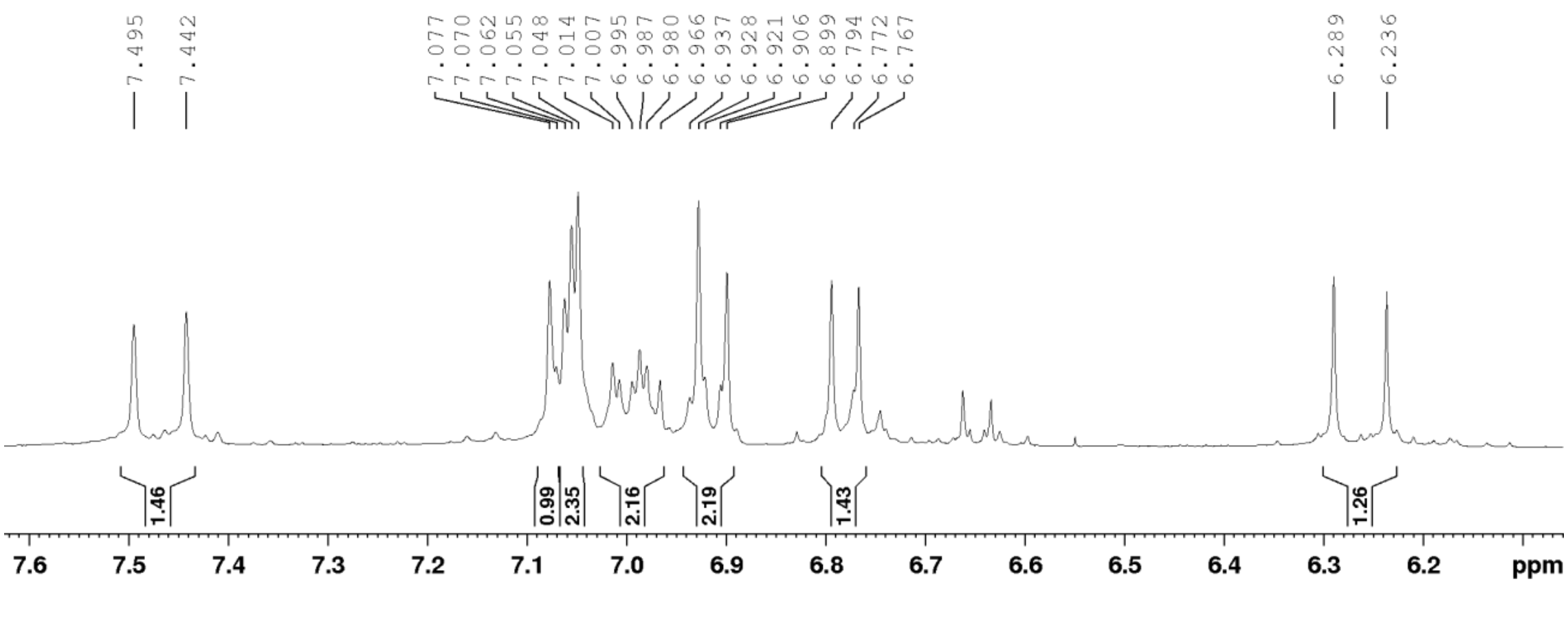

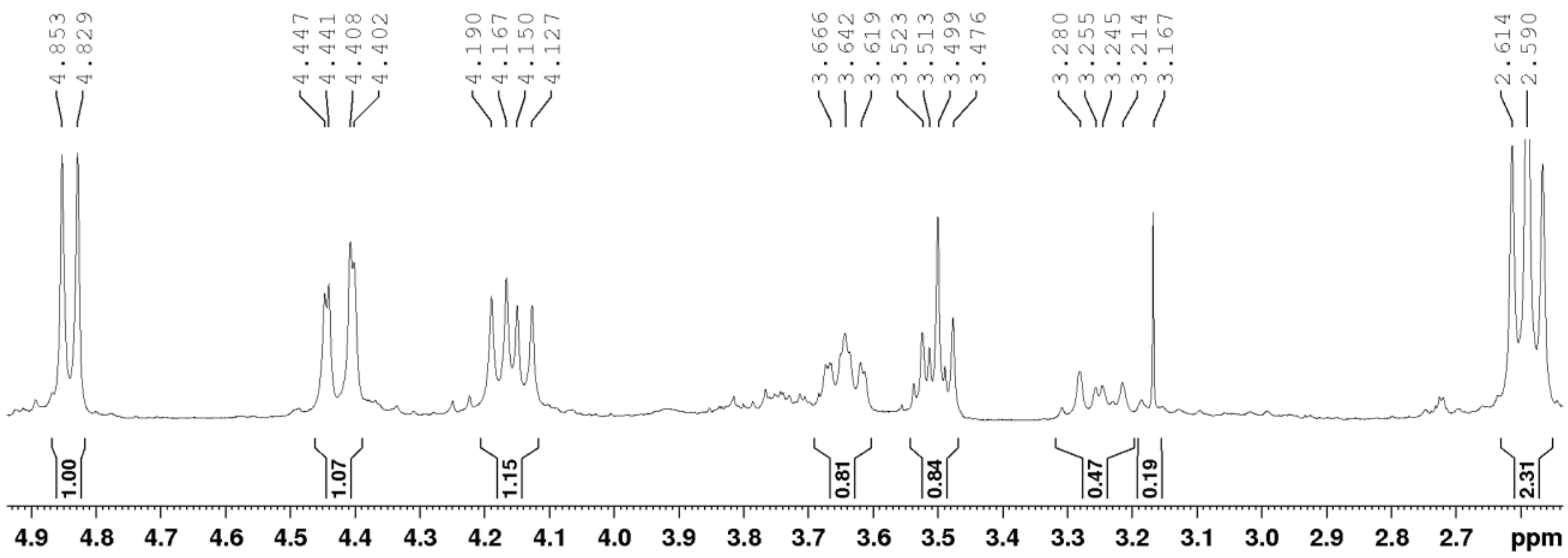

Note: The integrations in the sugar region (δ 3.2–3.8 ppm) are affected by water suppression and signal overlap.

Figure S13 - ^{13}C-NMR spectrum with expansion (DMSO-d_6, 75 MHz) of compound **8**.

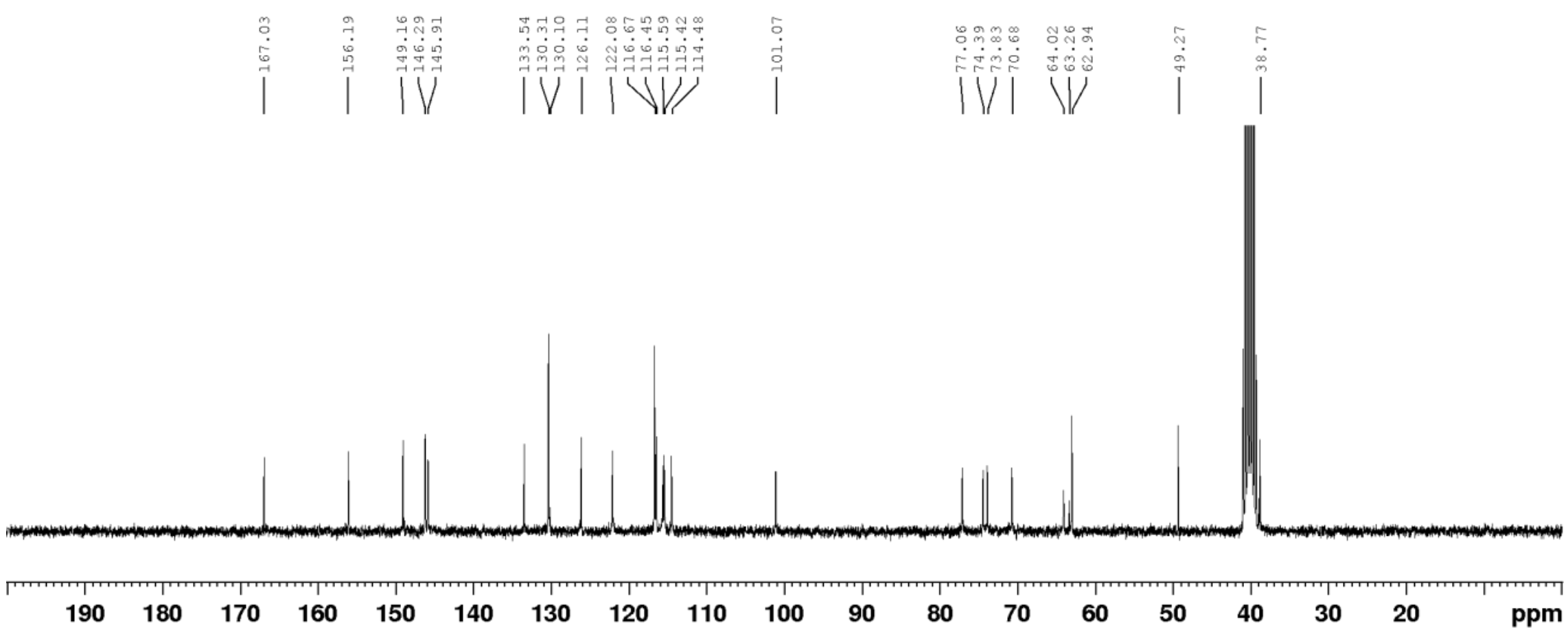

Figure S13 - ^{13}C-NMR spectrum with expansion (DMSO-d_6, 75 MHz) of compound **8** - continuation.

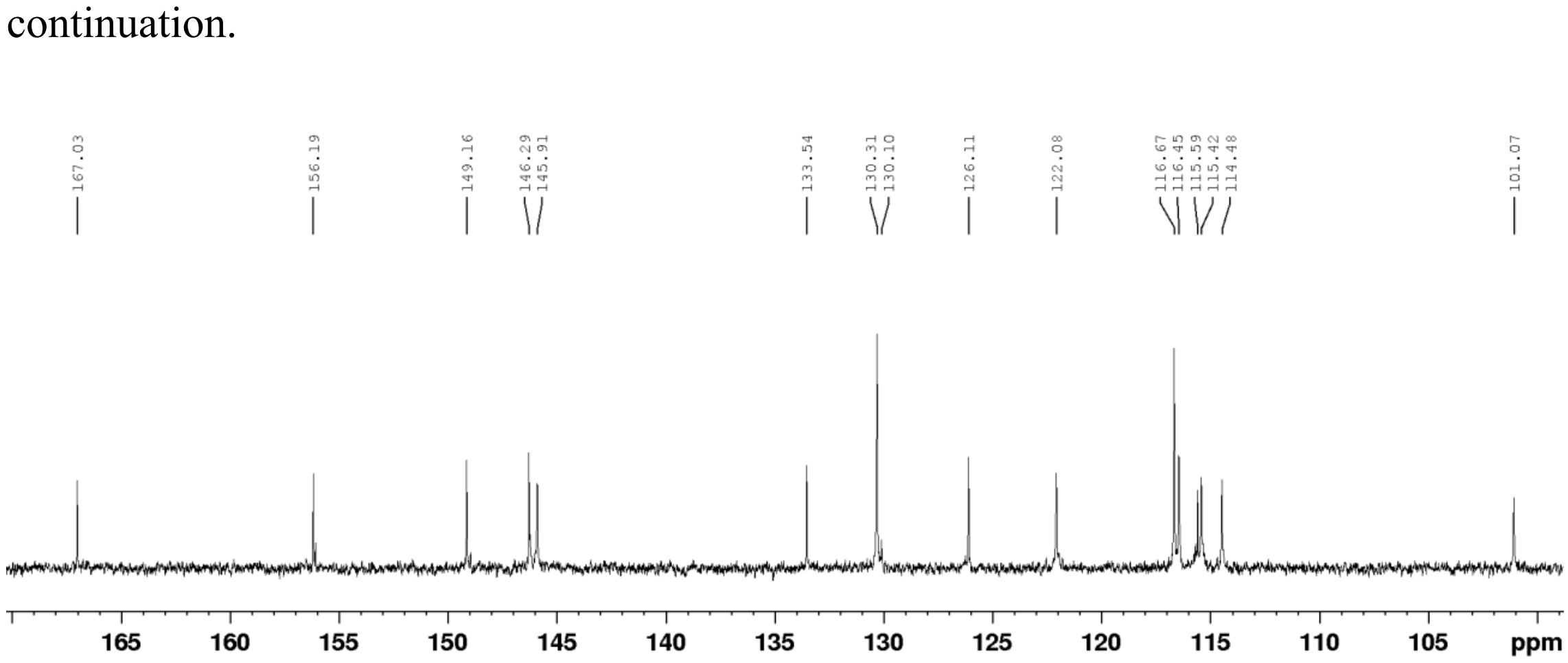

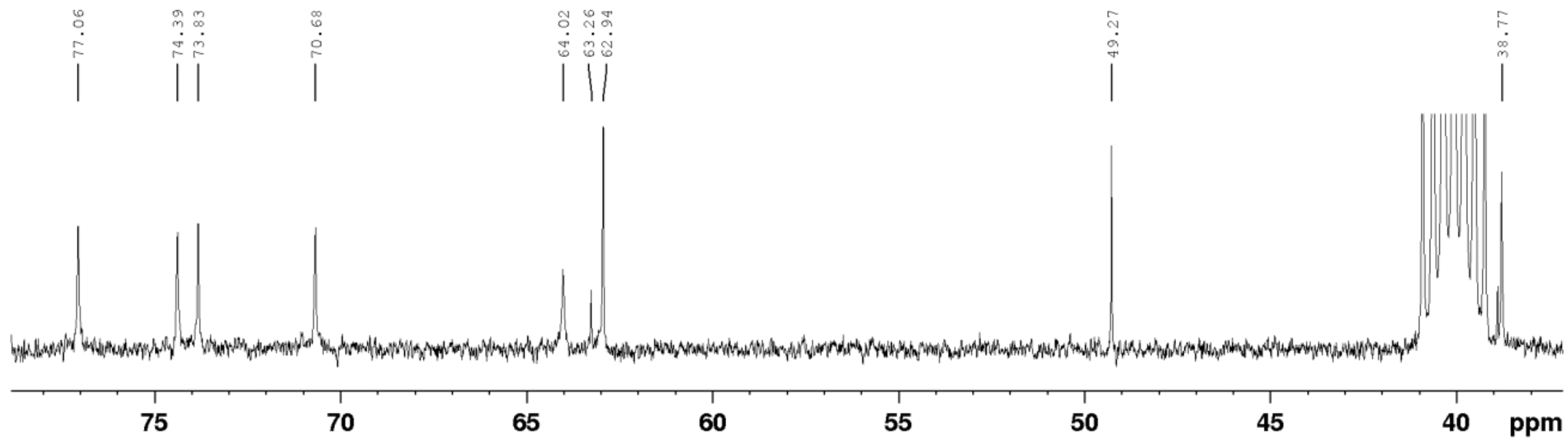

Figure S14 - ^{1}H-^{13}C HSQC spectrum (DMSO-d_6, 300 and 75 MHz) for **8**.

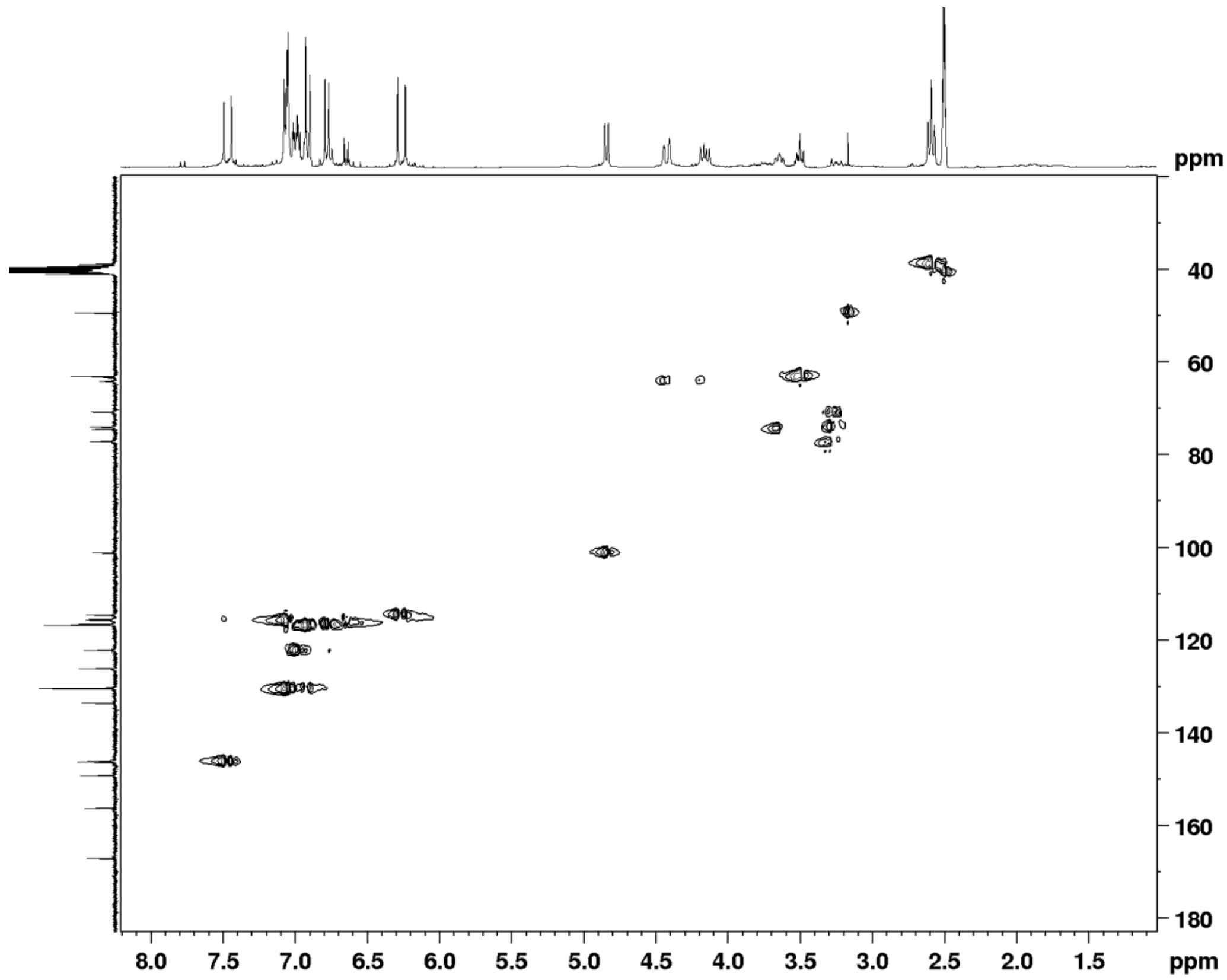

Figure S15 - ^{1}H-^{13}C HMBC spectrum with expansion (DMSO-d_6, 300 and 75 MHz) for **8**.

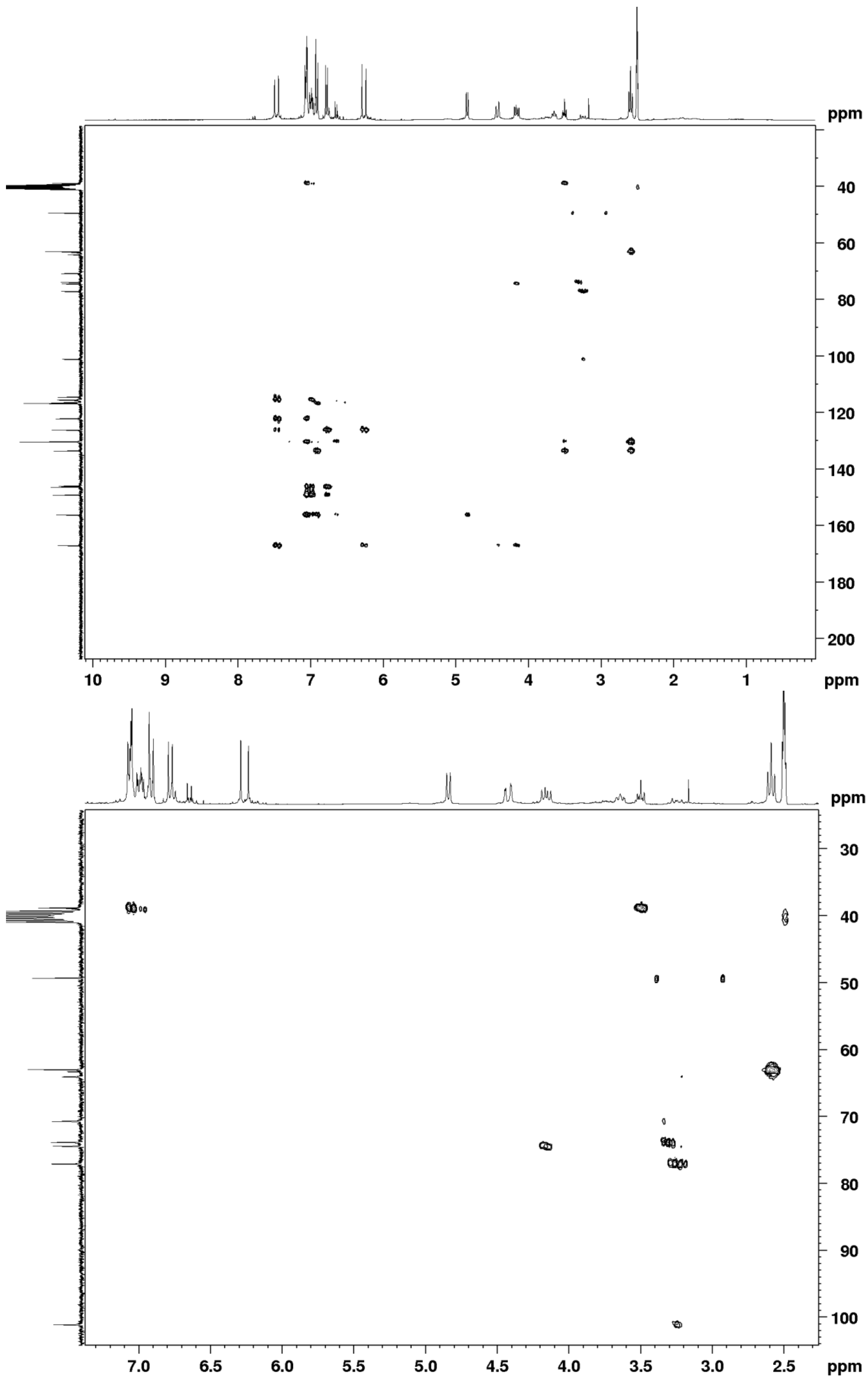

Figure S15 - ^{1}H-^{13}C HMBC spectrum with expansion (DMSO-d_6, 300 and 75 MHz) for **8** – continuation.

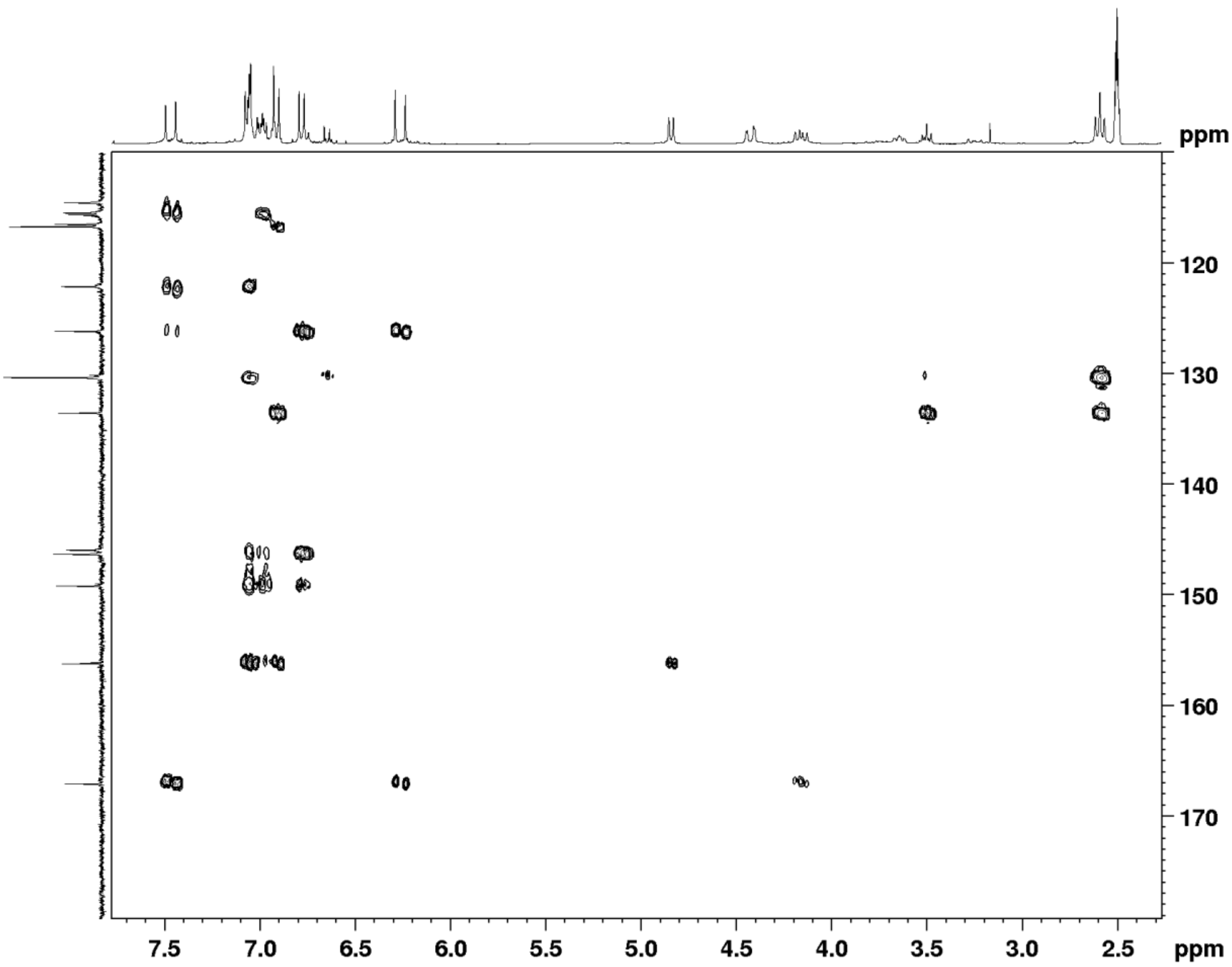

Figure S16 - COSY spectrum (DMSO-d_6. 300 MHz) of compound **8**.

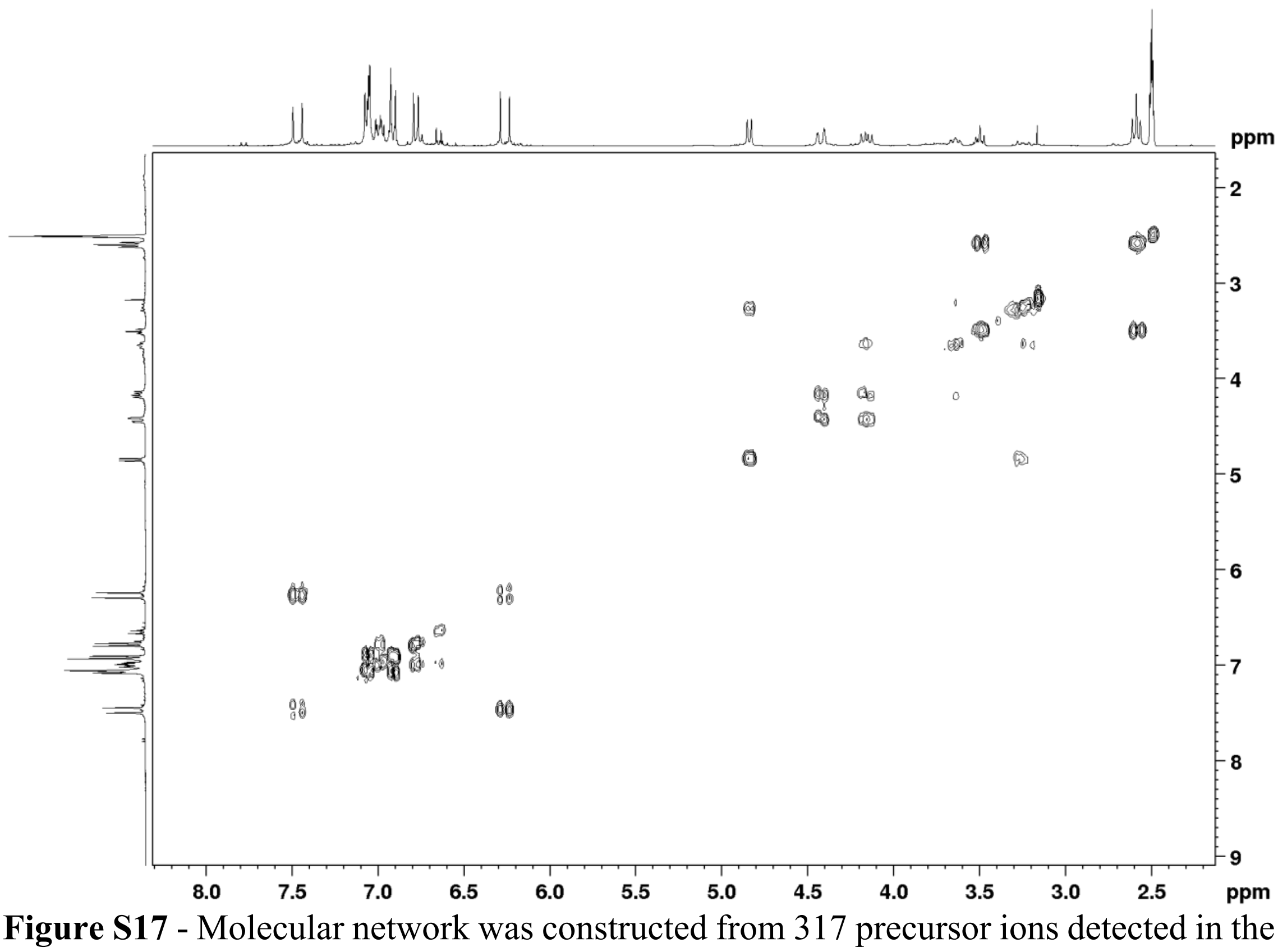

Figure S17 - Molecular network was constructed from 317 precursor ions detected in the negative ionization mode from UPLC-MS/MS data of *M. sordidum.* Nodes represent

detected compounds and are colored according to the respective fraction: green = crude ethanol extract (MSE), purple = ethyl acetate fraction (MSA), pink = dichloromethane fraction (MSD), and blue = hydromethanolic fraction (MSHM).

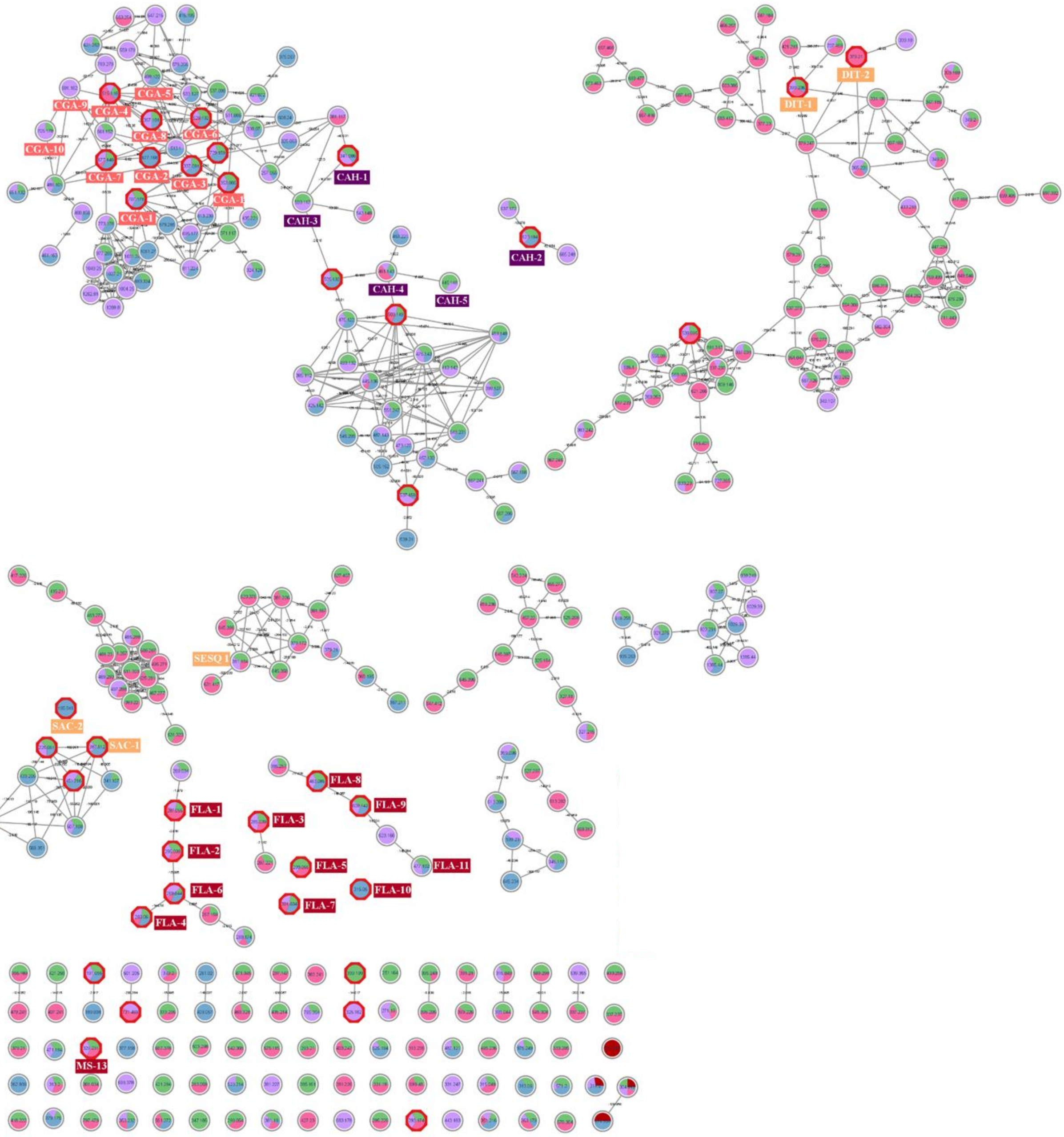

Antiproliferative assay:

Table S1. Inhibition rate (%) of samples submitted for testing against colon carcinoma cell line (HCT-116) at of 5 and 50 μg mL^{-1} concentrations. Data reflect the mean of three independent experiments ± SD and was determined by MTT assay after 72hs of incubation.

	**5 μg mL^{-1}**		**50 μg mL^{-1}**	
**Samples**	**% of cell growth inhibition**	**SD**	**% of cell growth inhibition**	**SD**
MSE	11.77	6.48	33.17	13.56
MSH	0	2.90	42.46	12.30
MSD	0	8.08	92.11	3.77
MSA	8.43	3.58	21.60	3.66
MSHM	14.48	6.27	7.41	9.75

MSE (crude extract); MSH (hexanic fraction); MSD (dichloromethane fraction); MSA (ethyl acetate fraction); MSHM (hydromethanolic fraction)
